# Early Growth Response Gene-2 Is Essential for M1 and M2 Macrophage Activation and Plasticity by Modulation of the Transcription Factor CEBPβ

**DOI:** 10.3389/fimmu.2018.02515

**Published:** 2018-11-01

**Authors:** Tatyana Veremeyko, Amanda W. Y. Yung, Daniel C. Anthony, Tatyana Strekalova, Eugene D. Ponomarev

**Affiliations:** ^1^Faculty of Medicine, School of Biomedical Sciences, The Chinese University of Hong Kong, Shatin, Hong Kong; ^2^Department of Pharmacology, University of Oxford, Oxford, United Kingdom; ^3^Department of Neuroscience, Maastricht University, Maastricht, Netherlands; ^4^Institute of General Pathology and Pathophysiology, Moscow, Russia; ^5^Laboratory of Psychiatric Neurobiology, Institute of Molecular Medicine and Department of Normal Physiology, Sechenov First Moscow State Medical University, Moscow, Russia; ^6^Kunming Institute of Zoology-Chinese University of Hong Kong Joint Laboratory of Bioresources and Molecular Research of Common Diseases, Kunming, China

**Keywords:** monocytes/macrophages, Egr2, CEBPβ, M1/M2 balance, activation, plasticity, inflammation

## Abstract

The process of macrophage polarization is involved in many pathologies such as anti-cancer immunity and autoimmune diseases. Polarized macrophages exhibit various levels of plasticity when M2/M(IL-4) macrophages are reprogrammed into an M1-like phenotype following treatment with IFNγ and/or LPS. At the same time, M1 macrophages are resistant to reprogramming in the presence of M2-like stimuli. The molecular mechanisms responsible for the macrophages polarization, plasticity of M2 macrophages, and lack of plasticity in M1 macrophages remain unknown. Here, we explored the role of Egr2 in the induction and maintenance of macrophage M1 and M2 polarization in the mouse *in vitro* and *in vivo* models of inflammation. Egr2 knockdown with siRNA treatment fail to upregulate either M1 or M2 markers upon stimulation, and the overexpression of Egr2 potentiated M1 or M2 marker expression following polarization. Polarisation with M2-like stimuli (IL-4 or IL-13) results in increased Egr2 expression, but macrophages stimulated with M1-like stimuli (IFNγ, LPS, IL-6, or TNF) exhibit a decrease in Egr2 expression. Egr2 was critical for the expression of transcription factors CEBPβ and PPARγ in M2 macrophages, and CEBPβ was highly expressed in M1-polarized macrophages. In siRNA knockdown studies the transcription factor CEBPβ was found to negatively regulate Egr2 expression and is likely to be responsible for the maintenance of the M1-like phenotype and lack plasticity. During thioglycolate-induced peritonitis, adoptively transferred macrophages with Egr2 knockdown failed to become activated as determined by upregulation of MHC class II and CD86. Thus, our study indicates that Egr2 expression is associated with the ability of unstimulated or M2 macrophages to respond to stimulation with inflammatory stimuli, while low levels of Egr2 expression is associated with non-responsiveness of macrophages to their activation.

## Introduction

Currently, it is accepted that at least two (among others) pathways for macrophage activation exist leading to two distinct or polarized states: the M1- and M2-like phenotypes (1, 2). Changes in M1 vs. M2 balance is a hallmark of many autoimmune diseases (2). Common autoimmune diseases such as multiple sclerosis and rheumatoid arthritis are associated with the presence of M1, M2 or mixed M1/M2 subsets with the predominant pathological role of an M1 subset (3, 4). The M1-like phenotype is mediated by Th1-associated cytokine IFNγ and microbial products such as LPS. M1-like macrophages express a high level of MHC class II and CD86 and effectively stimulate CD4 T cells to drive strong pro-inflammatory properties (2, 5, 6). These include the manufacture of NO (produced by enzyme NOS2) and the secretion of a number of potent pro-inflammatory cytokines such as IL-1β, IL-6, and TNF (2, 6). The M2-like macrophage phenotype is induced by the Th2-associated cytokines IL-4 or IL-13. M2 macrophages express low levels of CD86 and are poor stimulators of CD4 T cells, and seem to promote tissue repair during the resolution phase of inflammation (1, 7). Under normal conditions, most tissue-resident macrophages (e.g., peritoneal macrophages, microglia) exhibit M2-like characteristics. M2-like macrophages express a number of specific markers that include Arg1 (arginase), Fizz1 (cysteine-rich secreted protein), and Ym1 (extracellular matrix-binding lectin) (1, 8, 9). M2-like macrophages are known to produce more anti-inflammatory cytokines including IL-10 and TGFβ1; however, IL-10 is not considerate to be a specific M2 marker but is probably more indicative of the presence of deactivated macrophages (2, 10). The transcription factor PPARγ was recently shown to be involved in M2 polarization (6, 11), while its upstream transcription factor CEBPβ was shown to be necessary for the induction of both M1 and M2 markers upon stimulation with either M1 or M2 directing stimuli (2, 12–15).

Early growth response (Egr) proteins are a family of transcriptional regulators that mediate expression of multiple genes involved in cell growth and differentiation (16–18). There are four Egr proteins in the family and three (Egr1, Egr2, and Egr3) are expressed in macrophages (16, 19). The exact role of Egr1, Egr2, and Egr3 in the development and function of myeloid cells, such as monocytes and macrophages, remain unclear. It has been suggested that Egr1 plays an important role in the regulation of monocyte and macrophage differentiation. However, Egr1-deficient mice have normal numbers of macrophages, which suggests other functions for this protein must exist (19). Egr1, Egr2, and Egr3 expression was upregulated in myeloid progenitors when these cells are differentiated *in vitro* into macrophages in the presence of M-CSF (19). The role of Egr proteins was recently investigated in lymphoid cells (16); it was reported in this study that a conditional knockout for Egr2 and Egr3 resulted in a lethal autoimmune syndrome that was associated with excessive systemic levels of pro-inflammatory cytokines (20). The knockout also exhibited impaired antigen receptor-induced proliferation of B and T cells. It was suggested that Egr2 negatively regulates T and B cell activation and production of pro-inflammatory cytokines by the induction of suppressor of the cytokine signaling (SOCS) molecules SOCS1 and SOCS3 (20). In macrophages, Egr1 was shown to induce expression of SOCS1 in LPS-stimulated M1 macrophages (21). Similarly, Egr2 was found to be the positive regulator for SOCS1 and STAT5 in dendritic cells (22). In Tregs, Egr2 has been shown to regulate the expression of the anti-inflammatory cytokine TGFβ3 (23). In non-immune biology, Egr2 was found to be critical for hindbrain development and peripheral myelination and led to the perinatal death of Egr2-deficient mice (24, 25). However, the downstream action of Egr molecules in macrophages is still not well understood. Bone marrow-derived myeloid precursors from Egr1/Egr3-double knockout mice exhibited macrophage differentiation that is identical to that of wild-type mice (19). Moreover, fetal liver-derived myeloid precursors from Egr1/Egr2-double-deficient mice did not show abnormalities in macrophage differentiation (19). These data indicate that other functions of Egr-family proteins in myeloid cells exist beyond the development and differentiation of myeloid progenitors into monocytic cells. Drawing upon published results on both lymphoid and myeloid cells, we hypothesized, that Egr family proteins are likely to be involved in the control of macrophage activation and/or polarization.

Although polarized M1 and M2 macrophages exhibit distinct phenotypes *in vitro*, the picture is not so clear *in vivo* during inflammation, where macrophages often exhibit dually activated or a mixed M1/M2-like phenotype (7–9, 26). The mixed M1/M2-like phenotype was associated with a high level of macrophage plasticity: polarized macrophages seem to change their phenotype at a whole population-based level over the time (27). However, to date, it has not been clear how this process is regulated at a molecular level. There are several studies, including some of our own, that demonstrate how M2 macrophages could be switched to the M1-like phenotype *in vitro* and *in vivo* (28, 29). Much less is known about the potential to switch M1-like macrophages toward an M2-like phenotype. There is one study regarding the switch of human M1-like macrophages to M2, but his study was very restricted in terms of the choice of M1 and M2 markers and the M1-polarizing conditions were modest (30). It was concluded based on extensive literature analysis that it would not be common for M1 macrophages to be switched to M2 *in vivo* and that it would only happen in a mild inflammatory reaction (1). In our previous study, we found that IFNγ/LPS-treated M1-like macrophages were unresponsive to IL-4 and would not upregulate M2-associated molecules such as Mrc1 (CD206) and microRNA-124 (miR-124) and did not downregulate M1-associated miR-155, MHC class II and CD86 (28). The reason for such non-responsiveness of M1-like macrophages remained unexplained at a molecular level. One of the possible mechanisms is mitochondrial dysfunction of M1-like macrophages, but exact molecular pathways that lead to this state remained unknown (31).

Only recently Egr2 was proposed as a new M2 marker for macrophage alternative activation (32); however, exact functions of transcriptional regulator Egr2 in M1-like [M(IFNγ) and M(LPS)] and M2-like [M(IL-4) and M(IL-13)] macrophage polarization was not investigated in details. Since Egr2 was not involved in macrophage development, it was important to investigate the role of Egr2 in the ability of macrophages to become activated toward M1- or M2-like phenotypes and maintain their M1- or M2-like phenotype during their reprogramming. In contrast to myeloid dendritic cells, macrophages are quite a heterogeneous population and their mode of activation depends on local microenvironment leading to classic (M1-like) and alternative (M2-like) states. While Egr2 was found to be a negative regulator of activation of dendritic cells targeting SOCS1 (22), the role of Egr2 in macrophage activation is not clear. Therefore, it is very important to understand the role of Egr2 in macrophage activation.

In this study, we found that the Egr family proteins, Egr3 and Egr2, were differentially expressed in M1 and M2 macrophages. We established that although Egr2 was upregulated in M2 macrophages, the expression of this protein was required for upregulation of M1 or M2 markers in response to M1-like (IFNγ, LPS, TNF, IL-6) or M2-like (IL-4, IL-13) stimuli respectively. Treatment of macrophages with IFNγ and/or LPS resulted in long-term downregulation of Egr2, which lasted for more than 72 h. Low levels of Egr2 was correlated with non-responsiveness of M1 macrophages to further stimulation. Knockdown of Egr2 by siRNA decreased expression of the M2 markers Arg1, Ym1, Fizz1 and upregulated IL-10 in IL-4-treated macrophages. The introduction of Egr2 siRNA also made macrophages less responsive to IFNγ and/or LPS resulting in the downregulation of the M1-associated markers NOS2, TNF, IL-6, IL-1β and Ptgs2 (Cox-2) and upregulation of IL-10. Alternatively, overexpression of Egr2 increased expression of a number of M1- and M2-associated markers in response to IFNγ and IL-4 respectively. We also demonstrated that Egr2 positively regulated the expression of CEBPβ, while, in turn, Egr2 was negatively regulated by CEBPβ. Expression of Egr2 in macrophages appeared to be critical for upregulation of MHC class II and CD86 under inflammatory conditions. These data indicate a new role for Egr2 as a factor that positively regulates macrophage activation and plasticity during M1 and M2 polarization.

## Materials and methods

### Mice

C57BL/6 (B6) and C57BL/6-Tg(ACTB-bgeo/DsRed.MST (DsRed transgenic) mice were originally purchased from the Jackson Laboratory (Bar Harbor, ME) and bred locally at the Laboratory Animal Services Center (LASEC) at the Chinese University of Hong Kong. All animal procedures were conducted under individual licenses from the Hong Kong government and were approved by the animal ethics committee at the Chinese University of Hong Kong.

### Cells

Bone-marrow-derived macrophages (BMDMs) were grown in the presence of M-CSF as described earlier in our studies (28, 33). Bone marrow from 2 to 3 of 6 to 8 week-old B6 or DsRed transgenic mice was isolated by flushing from femur bones with PBS and a single cell suspension was obtained by passing the cell suspension through a 70 μm Cell Strainer (Falcon). Macrophages were grown in DMEM media (Gibco) supplemented with 10% FBS (Gibco) and 10 ng/ml M-CSF (R&D) for 5 days in 24-well plate (0.5 ml media/well). Media was replaced after every 2–3 days. After 5 days, the cells were used in experiments at the density of 300,000–400,000 cells per well, and the purity of the cells was more than 95% as determined by two-color flow cytometry analyzing expression of CD11b and F4/80. For macrophage polarization, the cells were treated with IL-4 or IL-13 (both from R&D, 50 ng/ml), or IFNγ (R&D, 100 ng/ml), or LPS (100 ng/ml; Sigma), or TNF (100 ng/ml), or IL-1β (100 ng/ml), or IL-6 (100 ng/ml), or GM-CSF (50 ng/ml) (all from R&D) for the periods indicated ranging from 2 to 24 h. The mouse macrophage cell line RAW264.7 was purchased from ATCC and maintained in DMEM media supplemented with 10% FBS.

### Flow cytometry

For analysis of cell surface markers, the cells were stained with anti-CD86-FITC or anti-CD86-PE, anti-MHC class II-PE-Cy5 (all from BD Biosciences), and F4/80-FITC (Biolegend) or F4/80-APC (eBioscience). Transfection plasmids and siRNA had fluorescent reporters (GFP for plasmid and Cy3-labeled RNA for siRNA), which were used to gate on transfected cells. Macrophages were analyzed for expression of surface markers (MHC class II, CD86, F4/80) or intracellular markers (Egr2, IL-6, TNF). FcRs were blocked with mAb specific for mouse FcR (2.4G2; BD Biosciences). Intracellular staining for Egr2, IL-6 or TNF was performed similarly as described earlier (32, 33) using fixation/permeabilization agent (eBioscience) and anti-Egr2-APC (eBioscience), anti-IL-6-PE, or anti-TNF-PE mAbs (both from BD Biosciences). The cells were analyzed using LSRFortressa^TM^ cytometer (BD Biosciences) and FlowJo software (Tree Star Inc.) as we described earlier (34).

### Quantitative real-time RT PCR

For quantitation by real-time RT PCR, total RNA was isolated by Qiagen or MirVana (Applied Biosystems) kits from BMDMs or RAW264.7 cell line. Real-time RT-PCR analyses were performed using TaqMan miRNA assays (Applied Biosystems). For analysis of mRNA expression of M1 and M2 associated molecules, we used primers that are described in Table 1. Relative expression levels were calculated using the ΔΔC_T_ method and normalized to the expression of GADPH housekeeping gene and then to the expression of a control sample that was defined as 1 (34). For the analysis of miR-155 expression, we used specific primers from Applied Biosystems and analyzed data by normalizing to the expression of short non-coding housekeeping snoRNA-55 and then to the reference sample using ΔΔC_T_ method as described previously (33, 35).

**Table 1:** Primer sequence for mRNA expression analysis.

**Gene**	**Primer**	**Sequence**
GADPH	Forward	5′-ATGACCACAGTCCATGCCATC-3′
	Reverse	5′-GAGCTTCCCGTTCAGCTCTG-3′
Arg1	Forward	5′CTTGGCTTGCTTCGGAACTC-3′
	Reverse	5′- GGAGAAGGCGTTTGCTTAGTTC-3′
CEBPβ	Forward	5′-GACAAGCTGAGCGACGAGTAC-3′
	Reverse	5′- TTGCGCATCTTGGCCTTGTC-3′
Cox-2/Ptgs2	Forward	5′-GTGACTGTACCCGGACTGGATTC-3′
	Reverse	5′- GGGTCAGGATGTAGTGCACTGTG-3′
Egr1	Forward	5′-GCAGCGCCTTCAATCCTCAAG-3′
	Reverse	5′-GCTCACGAGGCCACTGACTAG-3′
Egr2	Forward	5′- CCCTTTGACCAGATGAACGGAG-3′
	Reverse	5′-AAGCTACTCGGATACGGGAGATC-3′
Egr3	Forward	5′- CGACTCGGTAGCCCATTACAATC−3′
	Reverse	5′-GGGCTTCTCGTTGGTCAGAC-3′
Fizz1	Forward	5′-GCCAGGTCCTGGAACCTTTC-3′
	Reverse	5′-GGAGCAGGGAGATGCAGATGAG-3′
IL-1β	Forward	5′-CTTCCAGGATGAGGACATGAGCAC-3′
	Reverse	5′-TCATCATCCCATGAGTCACAGAGG-3′
IL-6	Forward	5′-CCTTCTTGGGACTGATGCTGGTG-3′
	Reverse	5′- AGGTCTGTTGGGAGTGGTATCCTC-3′
IL-10	Forward	5′-GGTTGCCAAGCCTTATCGGA-3′
	Reverse	5′-ACCTGCTCCACTGCCTTGCT-3′
NOS2	Forward	5′-ACCCACATCTGGCAGAATGAG-3′
	Reverse	5′-AGCCATGACCTTTCGCATTAG-3′
PPARγ	Forward	5′-AAGAGCTGACCCAATGGTTGC-3′
	Reverse	5′- AGGTGGAGATGCAGGTTCTACTTTG-3′
SOCS1	Forward	5′-CTCGTCCTCGTCTTCGTCCTC-3′
	Reverse	5′-GAAGGTGCGGAAGTGAGTGTC-3′
SOCS2	Forward	5′-ACCGACTAACCTGCGGATTGAG-3′
	Reverse	5′-CCTGTCCGTTTATCCTTGCACATC-3′
SOCS3	Forward	5′-GGCCACCTGGACTCCTATGAG-3′
	Reverse	5′-ACTGATCCAGGAACTCCCGAATG-3′
TNF	Forward	5′-AGCCGATGGGTTGTACCTTG-3′
	Reverse	5′- GTGGGTGAGGAGCACGTAGTC-3′
Ym1	Forward	5′-CCATTGGAGGATGGAAGTTTG-3′
	Reverse	5′- GACCCAGGGTACTGCCAGTC-3′

### Western blotting

Western blot analysis was performed according to a standard protocol as previously reported (33, 34). Antibodies for β-Actin (cat#4967), NOS2 (cat#9819), and Arg1 (cat#2982) were purchased from Cell Signaling. Antibodies for Egr2 (cat#692002) were purchased from Biolegend. Antibodies for CEBPβ (cat#606202) were purchased from Biolegend.

### Macrophage plasticity assay

Plasticity assay was performed as described earlier (28). BMDMs were polarized toward M1 with IFNγ (100 ng/ml) and LPS (100 ng/ml) or, toward M2 with IL-4 (50 ng/ml) for 24 h, before washing and culture in media alone (DMEM with 10% FBS and 10 ng/ml M-CSF) or they were further polarized in M1 (IFNγ, 100 ng/ml and LPS, 100 ng/ml) or M2 (IL-4, 50 ng/ml) for another 24-h period.

### Knockdown experiments using small interfering RNA

Egr2 interfering RNAs (siRNAs) (siGenome smart pool, cat# M-040303-01), and CEBPβ siRNA (siGenome smart pool, Cat # M-043110-02), and CEBPα siRNA [(siGenome smart pool, Cat # M-040561-01) and control siRNA (siGenome non-targeting control pool), cat# D-0011206-13-05] were purchased from Dharmacon (Lafayette, CO, USA) and used similarly as described earlier in our studies (36). BMDMs were transfected with siRNAs using TransIT-X2 System as a transfecting agent (Madison, WI, USA) according to the manufacturer's instructions. The efficiency of transfection was 80–90% as determined by the expression of a fluorescent reporter (Cy3-labeled RNA) in F4/80-positive cells as determined by flow cytometry.

### Overexpression plasmids and experiments

Expression plasmid pMIG-Egr2 were generated as follows. The first fragment of Egr2 gene was obtained from plasmid mEgr2/LZRS (Addgene; plasmid #27784) by using enzyme digestion via two restriction sites XhoI and BglII. Another fragment of Egr2 was amplified from mouse DNA library using primers 5′-CCTGAACTGGACCACCTCTACTC-3′ and 5′-CAGCAGATCTCACGGTGTCCTGGTTC-3′. Full Egr2 gene was amplified using primers 5′-TGCTCTCGAGATGATGACCGCCAAGGCCG-3′ and 5′-CGTCTGAATTCTCACGGTGTCCTGGTTCGAGAG-3′ and introduced into plasmid pMIG via restriction sites XhoI and EcoRI and subsequent ligation. Expression plasmid pMIG-Cebpb was kindly provided by Prof. Thomas Graf (Centre for Genomic Regulation, Barcelona, Spain). The efficiency of transfection was 20–50% as determined by expression of GFP reporter in F4/80-positive RAW264.7 cells as determined by flow cytometry.

### Nitric oxide synthase activity assay

NOS activity in BMDM cell lysate was measured using a colorimetric assay kit from Abcam (cat#ab211083) according to manufacturer's instructions.

### Thioglycollate-induced *in vivo* inflammation

Thioglycollate-induced peritonitis was initiated in the group of 4–5 B6 mice by i.p. injection of 2 ml of 4% thioglycollate broth media (Sigma) in PBS. The cells were isolated via peritoneal lavage 4 days after injection of thioglycollate media. Adoptive transfer of BMDMs transfected with Egr2 siRNA or Control miRNA was performed 30 min prior to administration of thioglycolate medium. BMDMs were grown in the presence of M-CSF from bone marrow of DsRed transgenic mice, transfected as described above, and 4–5 × 10^6^ cells per recipient B6 mouse were injected i.p. For FACS analysis, the cells were stained for CD86-FITC, MHC class II-PE-Cy5 and F4/80-APC, and DsRed^+^F4/80^+^ gated cells were analyzed for the expression of MHC class II and CD86.

### Statistical analysis

The results are presented as a mean ± standard error (S.E.). Unpaired Student's *t*-tests were used to determine significance between two independent groups. *P-*values of less than 0.05 were considered to be significant. SigmaPlot software was used for the creation of the graphs and statistical analysis.

## Results

### EGR2 is upregulated only in M2 but not M1 macrophages

**Figure 1:**
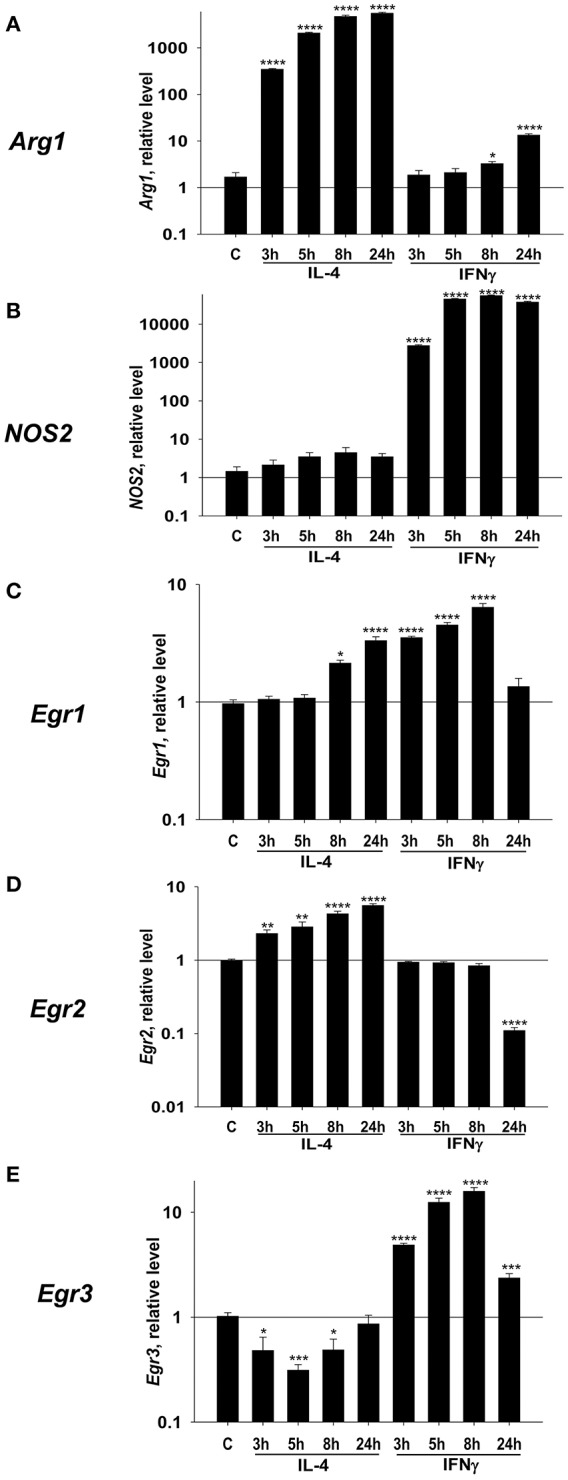
Kinetics of expression of Egr1, Egr2, and Egr3, M2 marker Arg1, and M1 marker NOS2 in macrophages polarized toward M2 (IL-4), or M1 (IFNγ). Bone-marrow-derived macrophages (BMDMs) were analyzed as untreated (C) or treated with IL-4 (IL4) or IFNγ (IFN) as described in *Materials and Methods*. The cells were analyzed after 3, 5, 8, and 24 h of incubation with indicated cytokines. For analysis, the cells were washed, mRNA was isolated and the expressions of *Arg1* **(A)**, *Nos2* **(B)**, *Egr1* **(C)**, *Egr2* **(D)**, and *Egr3* **(E)** were analyzed by real-time RT PCR as described in *Materials and Methods*. Mean ± S.E. of 12 separate culture plate wells is shown [^*^p < 0.05; ^**^p < 0.01; ^***^p < 0.001; ^****^p < 0.0001 when compared to untreated (C) cells].

In this study, we hypothesized that Egr1, Egr2, and Egr3 play an important role in the regulation of macrophage activation toward M1- and/or M2-like phenotypes. In the first series of experiments, we aimed to verify our hypothesis by investigating the kinetics of expression of these three genes in M2 and M1 polarized conditions. We used IL-4 or IFNγ to polarize macrophages toward M2 or M1, respectively. When we monitored expression of the M2 marker *Arg1* in IL-4 treated macrophages, we found it was upregulated as early as 3 h after treatment and peaked at 24 h. Conversely, IFNγ treatment did not upregulate Arg1 with the 3–24 h period, which indicated successful M2 polarization (Figure 1A). The M1 marker *NOS2* was not upregulated by IL-4, but it was upregulated by IFNγ as early as 3 h, peaked at 8 h, and was slightly decreased by 24 h (Figure 1B).

Next, we investigated the level of expression of *Egr1* and found that it was upregulated by both IL-4, and IFNγ (Figure 1C). *Egr2* was upregulated by IL-4, which is remained high for 24 h. By contrast, IFNγ treatment did not upregulate *Egr2* (Figure 1D). *Egr3* was only transiently upregulated by IFNγ that peaked at 8 h (Figure 1E). Thus, we found that all three members of Egr family were differentially regulated in macrophages following M1 or M2 polarization; Egr2 is associated with M2, Egr3 with M1-like macrophages, and Egr1 is a polarization marker (M0 to M1/M2).

### Egr2 is upregulated by IL-4 and IL-13 and downregulated by IFNγ, LPS, IL-6 and TNF

**Figure 2:**
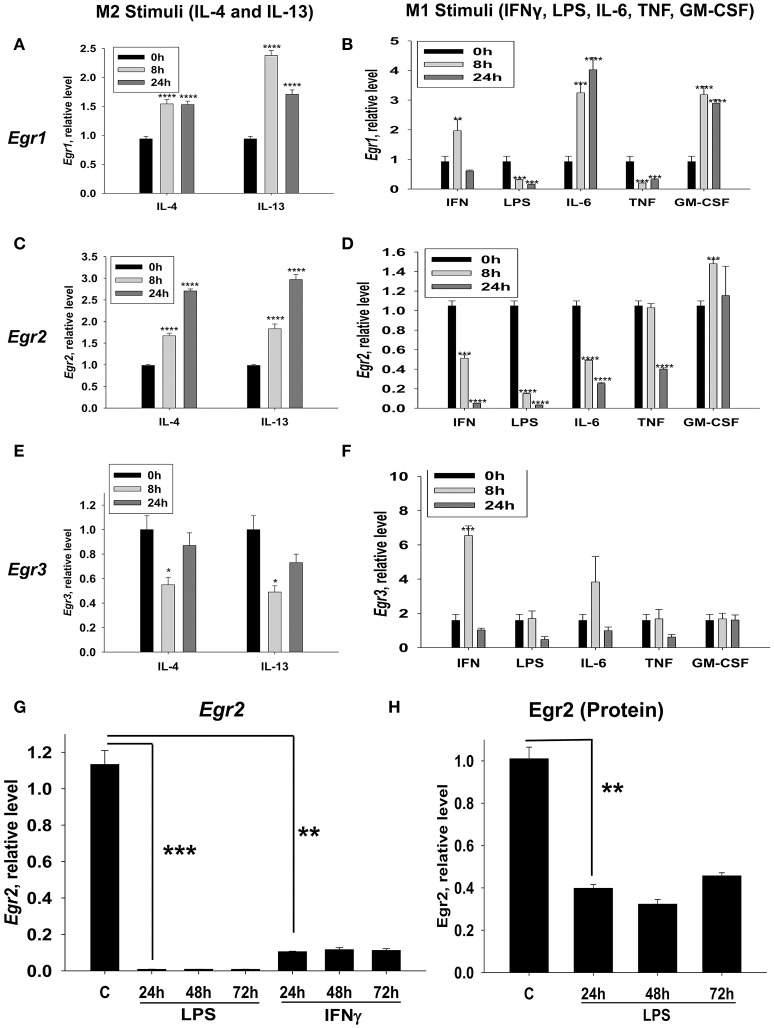
Kinetics of expression of Egr1, Egr2, and Egr3 in macrophages activated with various M2 (IL-4 and IL-13) and M1 (IFNγ, LPS, TNF, IL-6, and GM-CSF) stimuli. **(A–F)** Bone-marrow-derived macrophages (BMDMs) were treated with M2 stimuli IL-4 or IL-13 **(A,C,E)** or M1 stimuli IFNγ, LPS, TNF, IL-6, and GM-CSF **(B,D,F)** as described in *Materials and Methods*. The cells were analyzed immediately (0 h), or after 8 or 24 h of incubation with indicated M2 or M1 stimuli. For analysis, the cells were washed, mRNA was isolated and the expressions of *Egr1* **(A,B)**, *Egr2* **(C,D)**, and *Egr3* **(E,F)** were analyzed by real-time RT PCR as described in *Materials and Methods*. Mean ± S.E. of six separate culture plate wells is shown (^*^p < 0.05; ^**^p < 0.01; ^***^p < 0.001; ^****^p < 0.0001 when compared to 0 h cells). **(G)** BMDMs were treated with LPS or IFNγ as described in *Materials and Methods* and the cells were analyzed as untreated (C) or after 24 h, or 48 h, or 72 h of incubation with indicated M1 stimuli. For analysis, the cells were washed, mRNA was isolated and the expression of was analyzed by real-time RT PCR as described in *Materials and Methods*. Mean ± S.E. of six separate culture plate wells is shown (^**^p < 0.01; ^***^p < 0.001). **(H)** BMDMs were treated with LPS as described in *Materials and Methods* and the cells were analyzed as untreated (C) or 24 h, 48 h, and 72 h of incubation with LPS (100 ng/ml). For analysis, the cells were washed, and the expression of Egr2 was analyzed on a protein level by western blot as described in *Material and Methods*. β-Actin has used a loading control. Representative western blot is shown in Figure S1C. Mean ± S.E. of 3–4 separate culture plate wells is shown (^**^p < 0.01).

We investigated whether *Egr1, Egr2*, and *Egr3* were differentially regulated by M2-like stimuli (IL-4 and IL-13) and an expended set of M1-like stimuli (IFNγ, LPS, IL-6, IL-1, TNF, and GM-CSF). *Egr1* was upregulated by both M2-like stimuli IL-4 and IL-13 (Figure 2A) and a number of the M1-like stimuli (IFNγ, IL-6, and GM-CSF) (Figure 2B). However, *Egr1* was downregulated by LPS and TNF (Figure 2B). At the same time cell viability was not decreased in IFNγ, LPS, IL-6, or TNF treated macrophages as determined by vital dye staining (not shown). When we investigated *Egr2*, it was upregulated by IL-4 and IL-13 (Figure 2C) and it was downregulated by IFNγ, LPS, IL-6, and TNF, but not GM-CSF (Figure 2D). *Egr3* was transiently downregulated IL-4 and IL-13 (Figure 2E), while it was transiently upregulated by two M1-like stimuli IFNγ and IL-6 (Figure 2F). These data indicated that only *Egr2* was differentially regulated by M2- and most of the M1-like stimuli and mRNA expression of this molecule was stably high in M2 and stably low in M1 cells. Expression of Egr2 was also stably upregulated on protein level by IL-4 (Figures S1A,B), indicating a great importance of Egr2 vs. Egr1/2 in control of macrophages activation toward M2. Thus, we established that *Egr2* was upregulated by M2 stimuli IL-4 and IL-13 and downregulated by a number of M1 stimuli IFNγ, LPS, IL-6, and TNF.

### LPS and IFNγ cause long-term downregulation of Egr2 in M1 macrophages

Since Egr2 was differentially expressed in M1- and M2-like macrophages we focused on this factor and looked at the long-term kinetics of the expression of *Egr2* in M1-stimulated macrophages. We found that IFNγ- or LPS- treated macrophages displayed long-term downregulation of *Egr2*, which lasted for more than 72 h (Figure 2G). Downregulation of *Egr2* on mRNA level was also confirmed on a protein level indicating ~3-fold decrease in expression of this protein in M1 macrophages (Figure 2H, Figure S1C). Thus, M1 polarized macrophages downregulate Egr2 mRNA and protein levels, while Erg2 is upregulated in M2 macrophages.

### M1 macrophages exhibiting low levels of Egr2 fail to downregulate M1 markers and weakly upregulate M2 markers in response to treatment with IL-4

**Figure 3:**
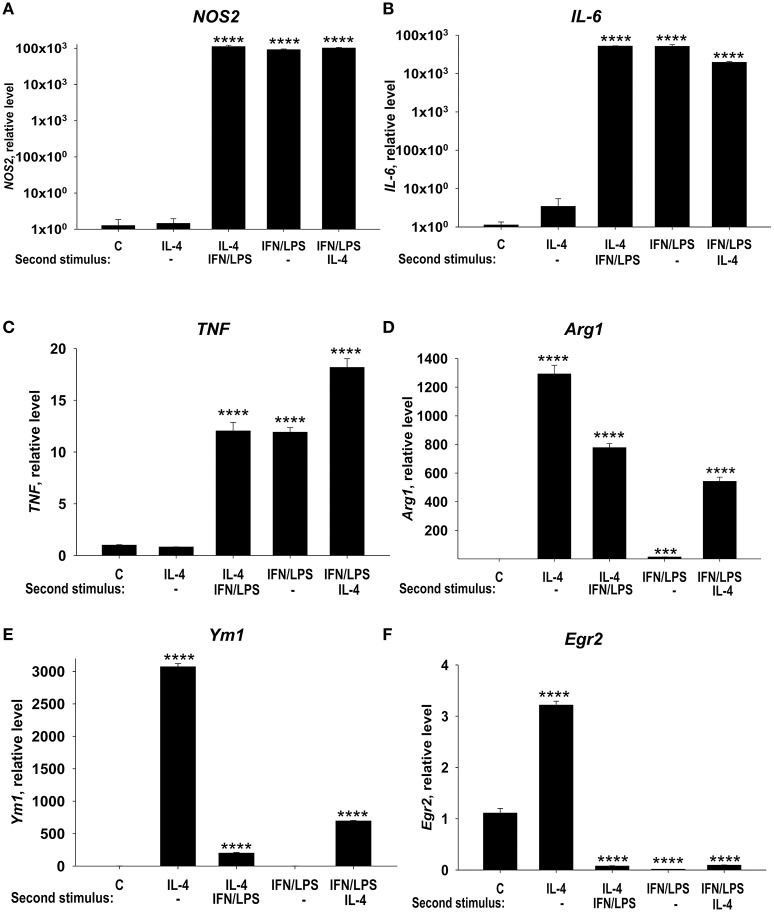
Analysis of expression of M1 markers (NOS, IL-6, and TNF), M2 markers (Arg1 and Ym1), and Egr2 in M1 macrophages that were subsequently stimulated toward M2 and vice versa. Bone-marrow-derived macrophages (BMDMs) were treated with M2 stimulus IL-4 or M1 stimulus IFNγ/LPS for 24 h, washed and then subjected to opposite M1 (IL-4) and M2 (IFNγ/LPS) stimuli for another 24 h-period as described in *Materials and Methods*. The cells were analyzed as unstimulated (C), or after 24 h of incubation with IL-4 only (IL4), or after incubation with IL-4 for 24 h, and then with IFNγ/LPS for 24 h (IL4 Second stimulus IFN/LPS), or after 24 h of incubation with IFNγ/LPS only (IFNγ/LPS), or after incubation with IFNγ/LPS for 24 h, and then with IL-4 for 24 h (IFN/LPS Second stimulus IL4). For analysis, the cells were washed, mRNA was isolated and the expressions of *NOS2* **(A)**, *TNF* **(B)**, *IL-6* **(C)**, *Arg1* **(D)**, *Ym1* **(E)**, and *Egr2* **(F)** were analyzed by real-time RT PCR as described in *Materials and Methods*. In **(A–F)**, mean ± S.E. of 4–6 separate culture plate wells is shown [^***^p < 0.001; ^****^p < 0.0001 when compared to unstimulated (C) cells].

In this study, we investigated the possibility to reprogram the M1- and M2-polarized macrophages. M1-like macrophages treated with IL-4 retained the capacity to express high levels M1 markers NOS2 (Figure 3A, *LPS/IFN*γ and *LPS/IFN*γ *Second stimulus: IL-4*), IL-6 (Figure 3B, *LPS/IFN*γ and *LPS/IFN*γ *Second stimulus: IL-4*), TNF (Figure 3C, *LPS/IFN*γ and *LPS/IFN*γ *Second stimulus: IL-4*), and displayed limited Arg1 induction (Figure 3D, *IL-4* and *LPS/IFN*γ *Second stimulus: IL-4*) or Ym1 induction (Figure 3E, *IL-4* and *LPS/IFN*γ *Second stimulus: IL-4*). Importantly the level of expression of Egr2 remained low/negative in M1 macrophages treated with IL-4 (Figure 3F, *LPS/IFN*γ, and *LPS/IFN*γ *Second stimulus: IL-4*). Conversely, M2 macrophages, which exhibited a high level of Egr2 expression (Figure 3F, *IL-4*), could switch and upregulated M1 markers NOS2, TNF, and IL-6 (Figures 3A–C, *IL-4 Second stimulus: LPS/IFN*γ) and downregulated M2 markers *Arg1, Ym1*, and *Egr2* (Figures 3D–F, *IL-4 Second stimulus: LPS/IFN*γ). These data were consistent with our previous study where we investigated surface markers MHC class II, CD86, and CD206 (28). Thus, our data demonstrate that M1 macrophages retained a low level of Egr2, which was associated with retention of M1 markers and weak upregulation of M2 markers.

### Knockdown of Egr2 resulted in a decrease in expression of M2 markers Arg1, Fizz1 and PPARγ and upregulation of IL-10 in IL-4-treated macrophages

**Figure 4:**
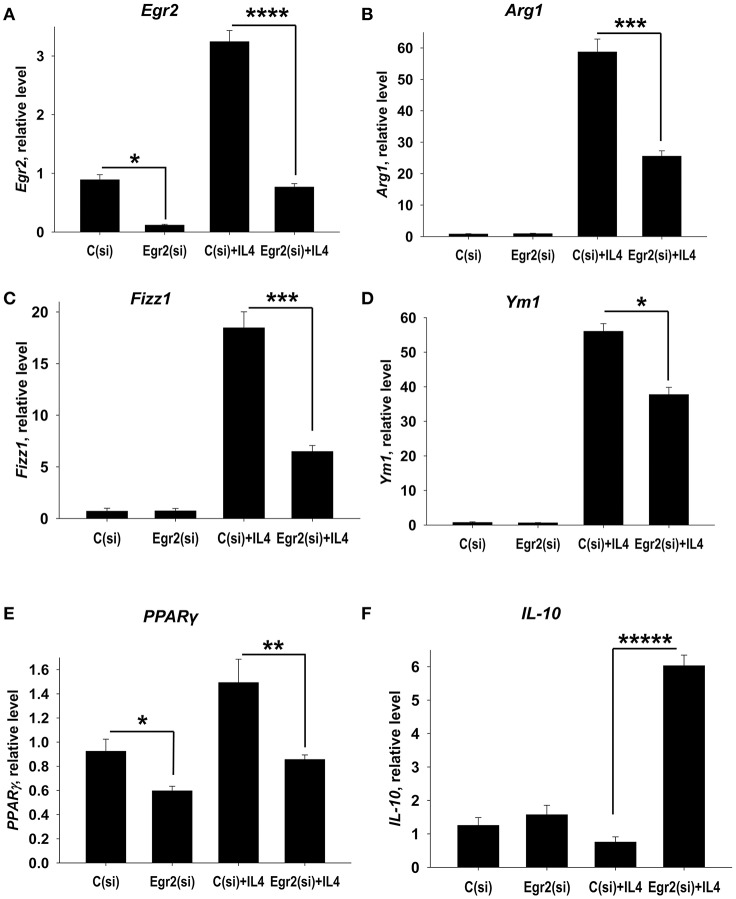
Analysis of expression of Egr2 and M2-associated markers (Arg1, Fizz1, Ym1, PPARγ) and IL-10 in IL-4-activated macrophages with knockdown of Egr2. Bone-marrow-derived macrophages (BMDMs) were transfected with siRNA cocktail for Egr2 [Egr2(si)] or control siRNA [C(si)] for 24 h as described in *Materials and Methods*, and after which the cells were used as unstimulated [C(si) and Egr2(si)] or activated with IL-4 for another 24 h-time period [C(si)+IL4 and Egr2(si)+IL4] as in Figure 3. The cells were washed, mRNA was isolated and the expressions of *Egr2* **(A)**, *Arg1* **(B)**, *Fizz1* **(C)**, *Ym1* **(D)**, *PPAR*γ **(E)**, and *IL-10* **(F)** were analyzed by real-time PCR as described in *Materials and Methods*. In **(A–F)**, mean ± S.E. of 4–9 separate culture plate wells is shown (^*^p < 0.05; ^**^p < 0.01; ^***^p < 0.001; ^****^p < 0.0001; ^*****^p < 0.00001).

We hypothesized that Egr2 may be essential for M2 polarization. To test this we knocked down Egr2 using siRNA technology. We found that the siRNA treatment markedly decreased expression of *Egr2* in unmanipulated and in IL-4-stimulated macrophages on mRNA (Figure 4A) and protein (Figures S2, S3) levels. We found that knockdown of Egr2 decreased the expression of *Arg1* (Figure 4B), *Fizz1* (Figure 4C), *Ym1* (Figure 4D), and *PPAR*γ (Figure 4E). The expression of IL-10 was significantly increased (Figure 4F). Thus, these results indicate that Egr2 promotes expression of the M2 markers Arg1, Fizz1, Ym1, and PPARγ and inhibits the expression of IL-10.

### Knockdown of Egr2 resulted in a decrease in the expression of M1 markers NOS2, Cox-2, TNF, IL-1β in IFNγ-treated macrophages

**Figure 5:**
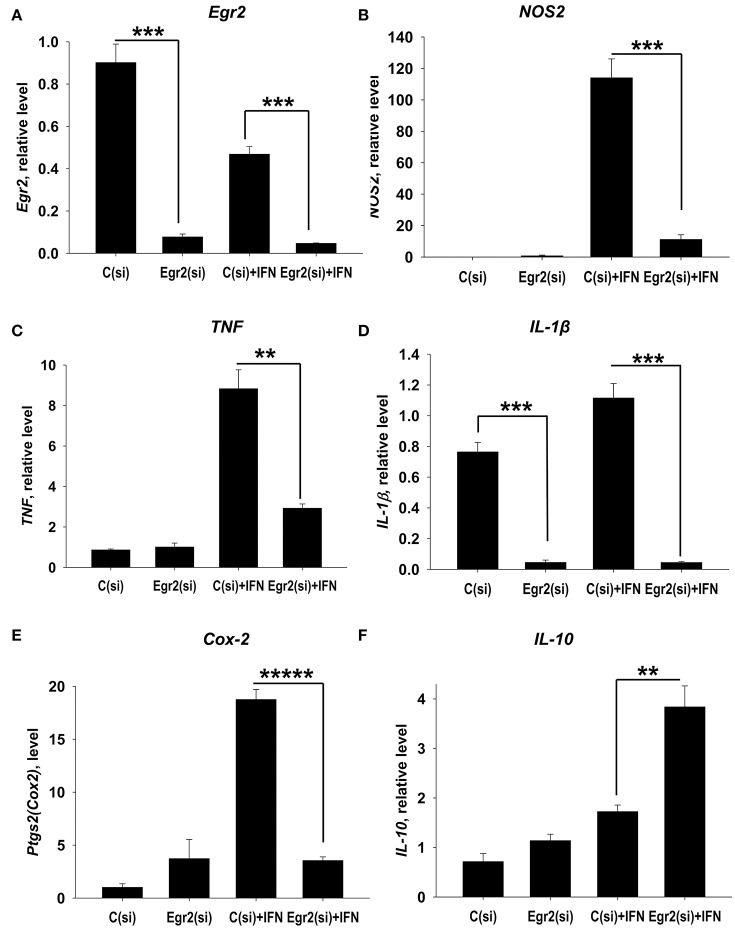
Analysis of expression of Egr2 and M1-associated markers (NOS2, TNF, IL-1β, Cox-2) and IL-10 in IFNγ-activated macrophages with knockdown of Egr2. Bone-marrow-derived macrophages (BMDMs) were transfected with siRNA cocktail for Egr2 [Egr2(si)] or control siRNA [C(si)] for 24 h as described in *Materials and Methods*, and after which the cells were used as unstimulated [C(si) and Egr2(si)] or activated with IFNγ for another 24 h-time period [C(si)+IFN and Egr2(si)+IFN] as in Figure 3. The cells were washed, mRNA was isolated and the expressions of *Egr2* **(A)**, *NOS2* **(B)**, *TNF* **(C)**, *IL-1*β **(D)**, *Cox-2* **(E)** and *IL-10* **(F)** were analyzed by real-time PCR as described in *Materials and Methods* similar as for Figure 2. In **(A–F)**, mean ± S.E. of six separate culture plate wells is shown (^**^p < 0.01; ^***^p < 0.001; ^*****^p < 0.00001).

We further examined the role of Egr2 in M1 polarization. Again, we found that siRNA for Egr2 substantially decreased expression of mRNA for Egr2 in IFNγ-stimulated M1 macrophages (Figure 5A). We found that knockdown of Egr2 significantly decreased expression of M1 markers *NOS2* (Figure 5B), *IL-1*β (Figure 5C), *TNF* (Figure 5D) and the activation marker *Cox-2(Ptgs2)* (Figure 5E). Surprisingly, as in M2-like macrophages, Egr2 knockdown in M1-like macrophages (Figure 4F) resulted in an increase in the expression of IL-10 (Figure 5F). The expression of M1-associated microRNA miR-155 was also inhibited in IFNγ-stimulated macrophages (Figure S4), further demonstrating the importance of Erg2 in the induction of M1-associated regulatory RNAs. Thus, these results indicate that Egr2 promotes expression of RNA of M1-associated molecules *NOS2, Cox-2, TNF, IL-1*β, and miR-155.

### Knockdown of Egr2 resulted in a decrease in the expression of M1 markers NOS2, TNF, IL-6, CD86, MHC class II, and M2 marker Arg1 on a protein level

**Figure 6:**
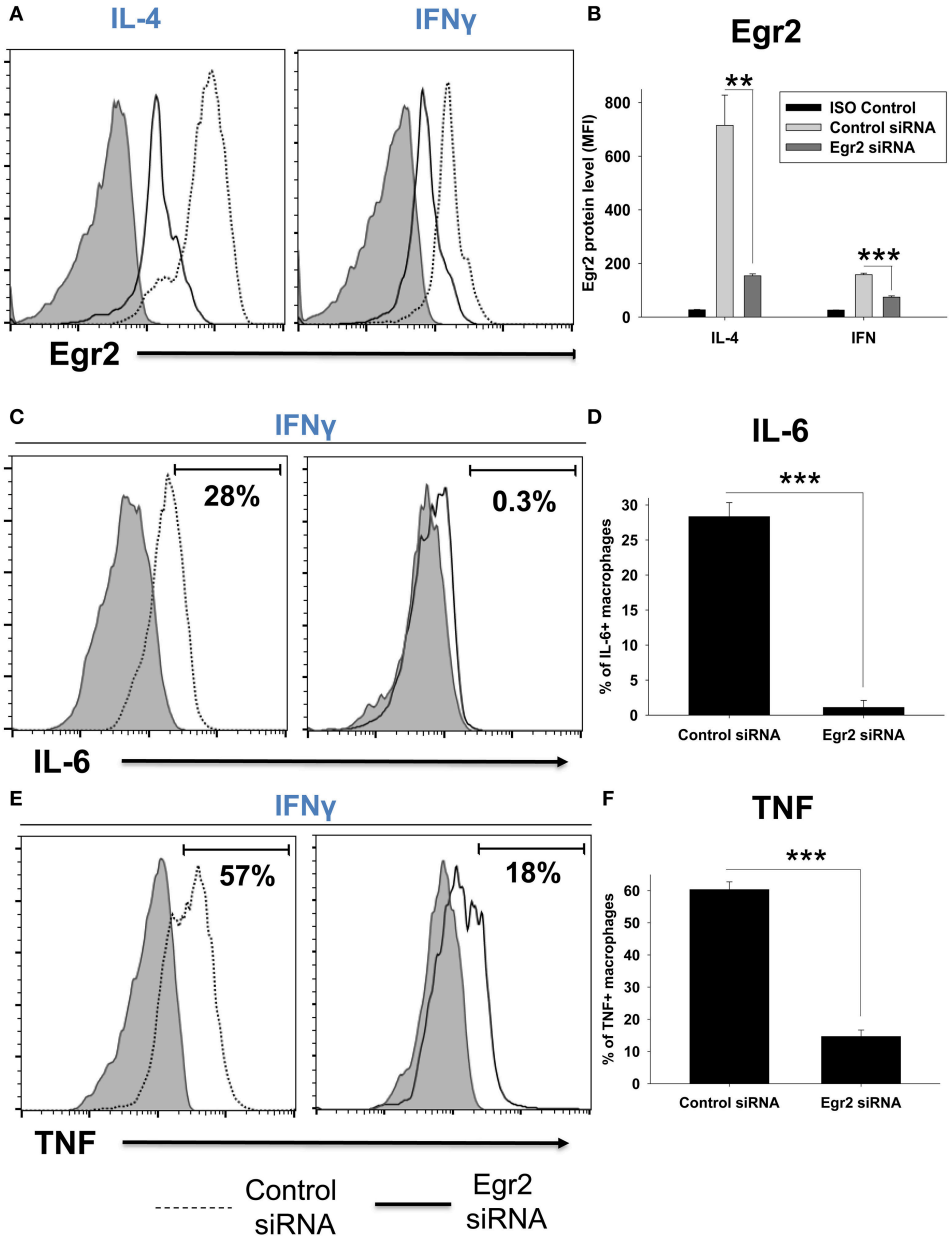
Analysis of expression of Egr2 on a protein level in M1- and M2-like macrophages with knockdown of Egr2 and intracellular cytokine protein level of TNF and IL-6 in IFNγ-activated macrophages with knockdown of Egr2. Bone-marrow-derived macrophages (BMDMs) were transfected with Egr2 siRNA or Control siRNA for 24 h as described in *Materials and Methods*, and after which the cells were activated with IL-4 or IFNγ for another 24 h-time period as in Figure 5. The cells were washed, stained for surface F4/80, fixed/permeabilized and stained for intracellular Egr2, IL-6, or TNF with fluorescently labeled mAbs and F4/80^+^ cells were analyzed by three-color flow cytometry as described in *Materials and Methods*. Expression of Egr2 **(A,B)**, IL-6 **(C,D)** and TNF **(E,F)** in macrophages transfected with Egr2 siRNA (solid line) vs. Control siRNA (dotted line) are shown on representative histogram graphs **(A,C,D)**. Staining with isotype-matched control mAbs (Control ISO) is shown by shaded histograms. Quantitative analysis (mean fluorescence intensity level for Egr2 and percentage of positive cells for IL-6 and TNF) is shown in **(B,D,F)**. In **(B,D,E)**, mean ± S.E. of six separate culture plate wells is shown (^**^p < 0.01; ^***^p < 0.001).

We further validated the decrease in expression of Egr2, IL-6, and TNF in macrophages on a protein level using intracellular staining and quantitative FACS analysis. We found that IL-4-treated macrophages expressed a high level of Egr2, which was significantly decreased by Egr2 siRNA (Figures 6A,B). When compared to IL-4-treated cells, IFNγ-treated macrophages expressed a ~10-fold lower level of Egr2, which was further decreased by Egr2 siRNA (Figures 6A,B). Knockdown of Egr2 in IFNγ-treated macrophages significantly decreased the production of M1 cytokines IL-6 and TNF on a protein level (Figures 6C–F).

**Figure 7:**
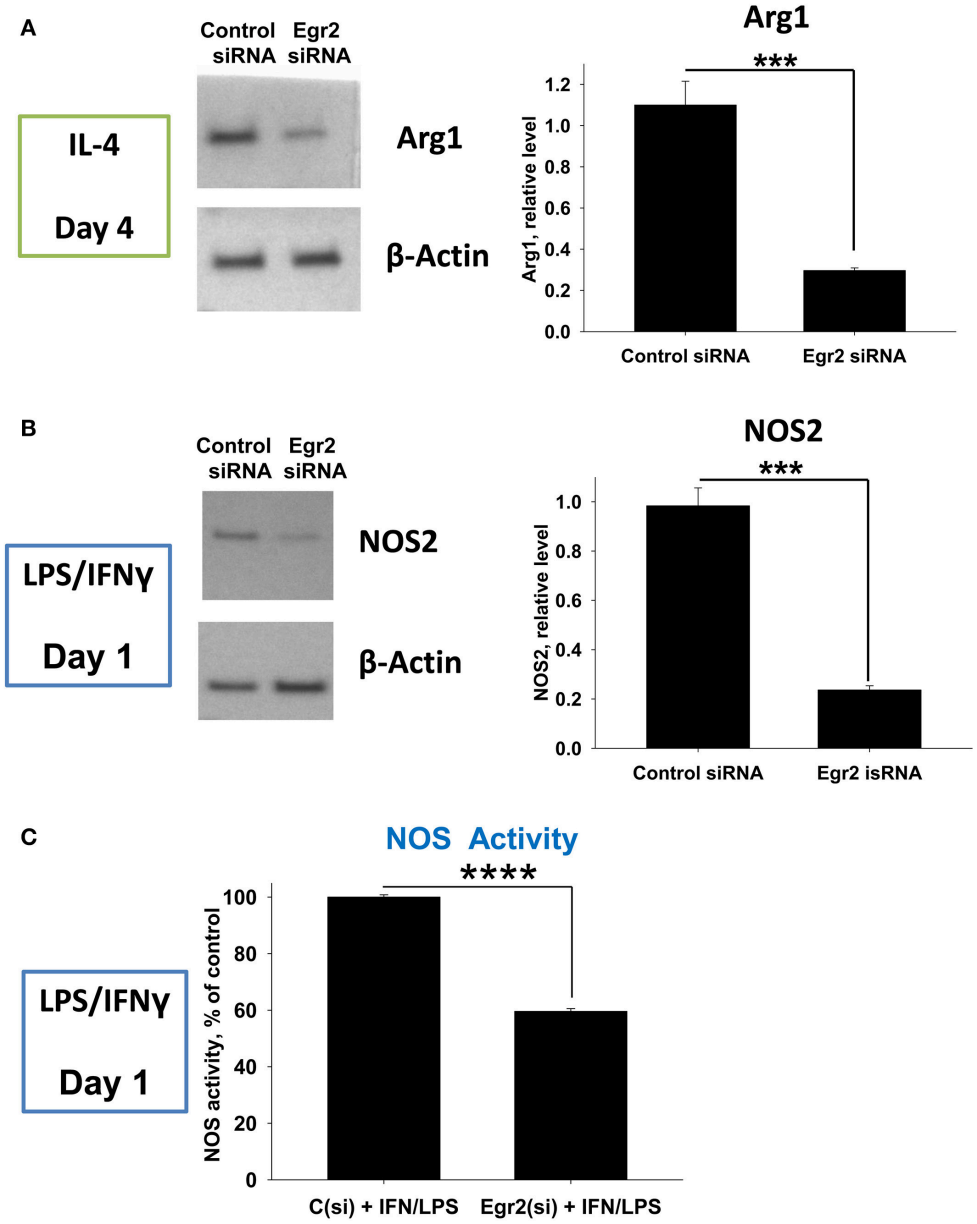
Analysis of the expression of Arg1 on a protein level in M2 macrophages with knockdown of Egr2 and NOS2 protein expression level along with NOS enzymatic activity in M1 macrophages with knockdown of Egr2. Bone-marrow-derived macrophages (BMDMs) were transfected with Egr2 siRNA [Egr2(si)] or Control siRNA [C(si)] for 24 h as described in *Materials and Methods*, and after which the cells were treated with IL-4 or IFNγ/LPS as described in *Materials and Methods* and the cells were analyzed after 96 or 24 h of incubation with IL-4 or IFNγ/LPS, respectively. For the analysis, the cells were washed, and the expression of Arg1 **(A)** or NOS2 **(B)** was analyzed on a protein level by western blot as described in *Material and Methods*. β-Actin has used a loading control. Representative blots are shown on the left and quantitative analysis is shown on the right. NOS enzymatic activity was measured as described in *Material and Methods* is shown for IFNγ/LPS-treated macrophages transfected with Egr2 siRNA vs. Control siRNA **(C)**. In **(A–C)**, mean ± S.E. of quadruplicate is shown. Statistically significant differences in the expression levels with are shown on the figures (^***^p < 0.001; ^****^p < 0.001).

Next, we validated downregulation of Arg1 in M2-like and NOS2 in M1-like macrophages on a protein level. We found that expression of Arg1 was not detected in IL-4-treated macrophages on a protein level on day 1, but appeared on day 4 post-IL-4 treatment (not shown). Knockdown of Egr2 significantly decreased the expression of Arg1 on a protein level on day 4 (Figure 7A). Expression of NOS2 was detected in M1 macrophages on day 1 post-IFNγ treatment and expression of this protein was significantly decreased by Egr2 siRNA (Figure 7B). NOS activity was also decreased in M1-like macrophages with an Egr2 knockdown, as was determined by actual NO production (Figure 7C).

**Figure 8:**
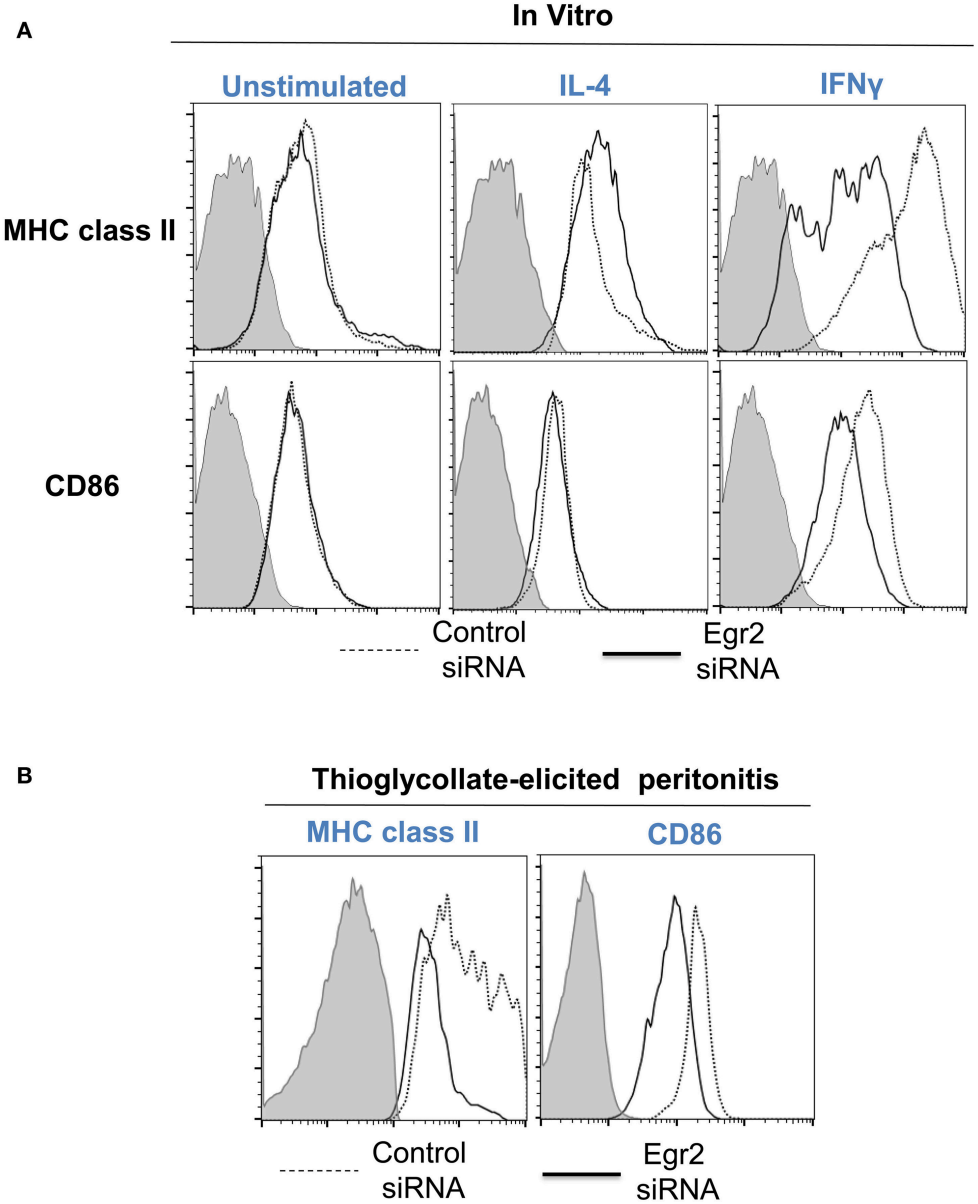
Analysis of expression of activation surface markers MHC class II and CD86 on macrophages with knockdown of Egr2 under inflammatory conditions *in vitro* and *in vivo*. Bone-marrow-derived macrophages (BMDMs) from B6 **(A)** or DsRed transgenic **(B)** mice were transfected with Egr2 siRNA or Control siRNA for 24 h, and after which the cells were used as unstimulated or activated *in vitro* with IL-4 or IFNγ for another 24 h-time period **(A)** or *in vivo* for 4 days in the model of thioglycollate-induced inflammation as described in *Materials and Methods*. **(A)** After *in vitro* incubation in media, IL-4 or IFNγ, the cells were washed, stained for surface markers F4/80, MHC class II and CD86 and F4/80^+^ gated macrophages were analyzed for the expression of MHC class II and CD86 by three-color follow cytometry as described in *Materials and Methods*. The expressions for MHC class II (top histograms) and CD86 (bottom histograms) of untreated (left histograms) or activated with IL-4 (middle histograms) or IFNγ (right histograms) macrophages transfected with Egr2 siRNA (solid line) vs. control siRNA (dotted line) are shown on representative histogram graphs. Staining with isotype-matched control mAbs is shown by shaded histograms. **(B)** The transfected DsRed-positive macrophages were injected i.p. into a group of 4-5 mice and peritoneal inflammation was induced by injection of thioglycollate medium as described in *Materials and Methods*. On day 4 after induction of inflammation, the cells were isolated by peritoneal lavage and cells were washed, stained for surface markers F4/80, MHC class II and CD86. F4/80^+^DsRed^+^ gated macrophages were analyzed for the expression of MHC class II and CD86 by four-color follow cytometry as described in *Materials and Methods*. The expressions for MHC class II (left histograms) and CD86 (right histograms) on F4/80^+^DsRed^+^ macrophages transfected with Egr2 siRNA (solid line) vs. control siRNA (dotted line) are shown on representative histogram graphs. Staining with isotype-matched control mAbs is shown by shaded histograms. **(C)** In **(A,B)**, quantifications and statistics are shown in Table 2.

Finally, we performed analysis of expression of classical macrophage activation marker MHC class II and M1-associated marker CD86 and found that both markers were downregulated by siRNA for Egr2 in IFNγ-treated M1 macrophages (Figure 8A). Quantification is shown in Table 2. At the same time, siRNA for Egr2 did not significantly affect the expression of these markers in M2-like macrophages (Table 2). Thus, we found that although Egr2 is upregulated in M2 macrophages, expression of Egr2 is also important for M1 polarization.

**Table 2:** Effect of Egr2 knockdown on expression of macrophage activation markers MHC class II and CD86 during inflammatory conditions^a^.

	**M0 (Unstimulated)**		**M2 (IL-4)**		**M1 (IFNγ)**		**Thioglycollate-induced inflammation**	
	**Control siRNA**	**Egr2 siRNA**	**Control siRNA**	**Egr2 siRNA**	**Control siRNA**	**Egr2 siRNA**	**Control siRNA**	**Egr2 siRNA**
MHC class II	257 ± 16	259 ± 27	282 ± 9	311 ± 7	611 ± 35	208 ± 39^b^	1836 ± 228	905 ± 86^c^
CD86	67 ± 7	74 ± 5	83 ± 8	99 ± 6	282 ± 8	170 ± 8^c^	173 ± 3	136 ± 10^d^

a *BMDMs were grown from bone marrow of B6 or DsRed transgenic mice in the presence of M-CSF for 5 days and transfected with Control siRNA or Egr2 siRNA. The cells were analyzed in vitro or injected i.p. into the group of 4–5 mice with thioglycollate-induced inflammation. For in vitro analysis, the cells were incubated in media alone (unstimulated) or in the presence of IL-4 (50 ng/ml) or IFNγ (100 ng/ml) as described in Materials and Methods. After 24 of incubation in vitro or on day 4 after induction of thioglycollate-elicited inflammation, the cells were stained for MHC class II, CD86, and F4/80. The F4/80^+^ gated (in vitro) or F4/80^+^DsRed^+^ (ex-vivo isolated) gated cells were analyzed for expression of MHC class II and CD86 by 3–4-color flow cytometry and mean fluorescence intensity (MFI) levels for expression of MHC class II and CD86 were measured. Mean ± S.E. of three separate experiments or 4–5 individual animals is shown*.

b *P < 0.001 when compared to control siRNA*.

c *P < 0.01 when compared to control siRNA*.

d *P < 0.01 when compared to control siRNA*.

### Knockdown of Egr2 resulted in a decrease in the expression of MHC class II and CD86 on macrophages during thioglycolate-induced inflammation of the peritoneum

We then tested whether Egr2 is important during inflammation *in vivo*. To test this, we used adoptively transferred bone-marrow derived macrophages (BMDMs) that expressed genetic markers DsRed under the actin promoter and were transfected with siRNA for Egr2 or Control siRNA. On day 4 of thioglycollate-induced peritonitis, adoptively transferred DsRed^+^F4/80^+^ macrophages transfected with control siRNA upregulated MHC class II and CD86, while macrophages with siRNA for Egr2 had a significantly lower level of expression of these molecules (Figure 8B). Quantification is shown in Table 2. Thus, we found that expression of Egr2 was important for macrophages activation *in vivo* during inflammation.

### Overexpression of Egr2 in IFNγ- and/or LPS-treated macrophages resulted in upregulation of M1 markers TNF, NOS2, IL-6, IL-1β, and Cox-2

**Figure 9:**
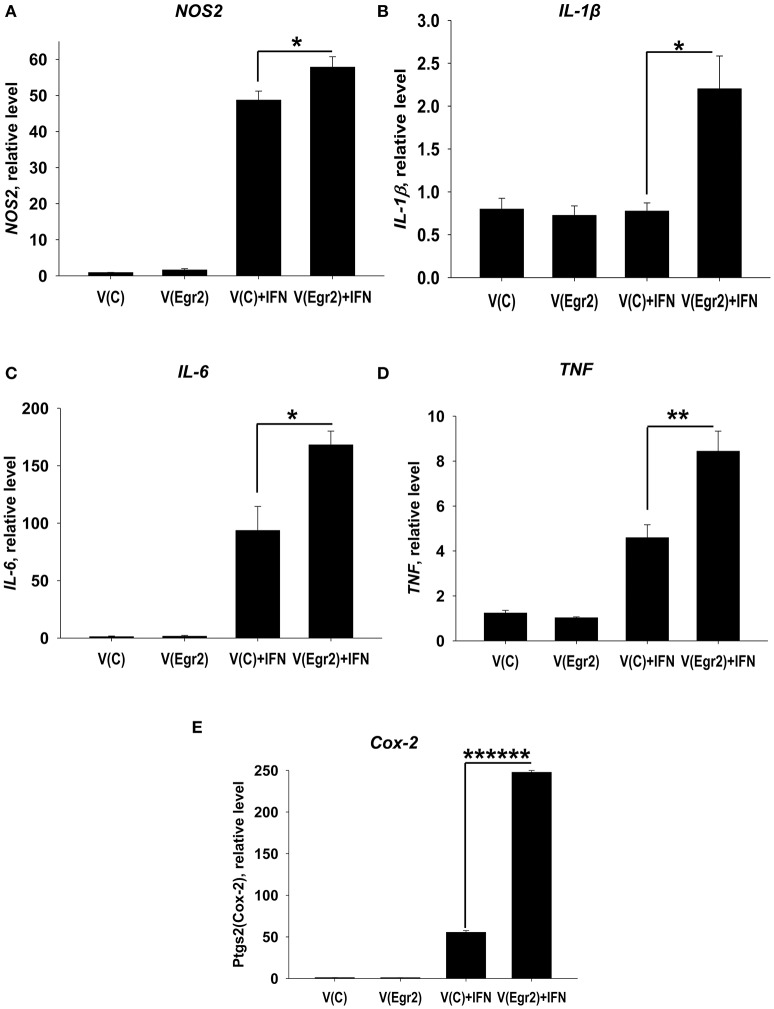
Analysis of expression of M1-associated markers (NOS2, IL-1β, IL-6, TNF, Cox-2) in IFNγ-activated macrophage cell line with overexpression of Egr2. Macrophage cell line RAW264.7 was transfected with empty (control) pMIG expression vector plasmid [V(C)] or vector with Egr2 [V(Egr2)] for 24 h as described in *Materials and Methods*. Then the cells were used as unstimulated [V(C) and V(Egr2)] or activated with IFNγ (100 ng/ml) for another 24 h-time period [V(C)+IFN and V(Egr2)+IFN]. The cells were washed, mRNA was isolated, and the expressions of *NOS2* **(A)**, *IL-1*β **(B)**, *IL-6* **(C)**, *TNF* **(D)**, *Cox-2* **(E)** were analyzed by real-time PCR as described in *Materials and Methods*. In **(A–E)**, mean ± S.E. of six separate culture plate wells is shown (^*^p < 0.05; ^**^p < 0.01; ^******^p < 0.000001).

**Figure 10:**
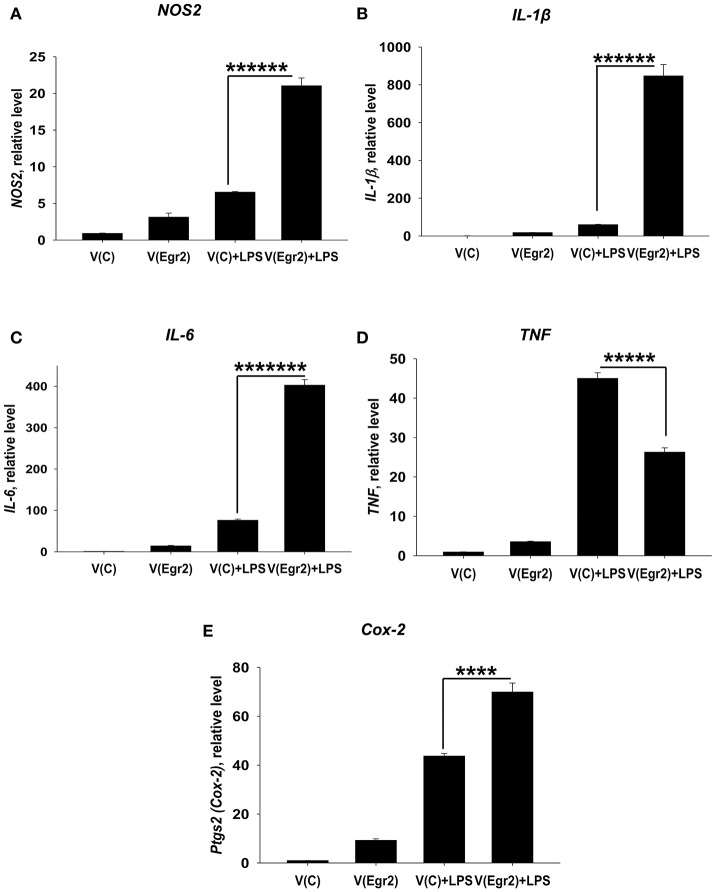
Analysis of expression of M1-associated markers (NOS2, IL-1β, IL-6, TNF, Cox-2) in LPS-activated macrophage cell line with overexpression of Egr2. Macrophage cell line RAW264.7 was transfected with empty (control) pMIG expression vector plasmid [V(C)] or vector with Egr2 [V(Egr2)] for 24 h and after which the cells were used as unstimulated [V(C) and V(Egr2)] or activated with LPS (100 ng/ml) for another 24 h-time period [V(C)+LPS and V(Egr2)+LPS] as in Figure 7. The cells were washed, mRNA was isolated and the expressions of *NOS2* **(A)**, *IL-1*β **(B)**, *IL-6* **(C)**, *TNF* **(D)**, *Cox-2* **(E)** were analyzed by real-time PCR as described in *Materials and Methods*. In **(A–E)**, mean ± S.E. of six separate culture plate wells is shown (^****^p < 0.0001; ^*****^p < 0.00001; ^******^p < 0.000001; ^*******^p < 0.0000001).

To confirm that expression of Egr2 is important for upregulation of M1-associated markers we overexpressed Egr2 in mouse macrophage cell line RAW264.7 as BMDMs are resistant to transfection with plasmids. We found that overexpression of Erg2 enhanced expression of *NOS2* (Figure 9A), *IL-1*β (Figure 9B), *IL-6* (Figure 9C) *TNF* (Figure 9D), and *Cox-2* (Figure 9E) in the macrophage cell line stimulated with IFNγ. Similarly, overexpression of Erg2 enhanced expression of *NOS2* (Figure 10A), *IL-1*β (Figure 10B), *IL-6* (Figure 10C), and *Cox-2* (Figure 10E) in the RAW264.7 cell line stimulated with LPS. However, the expression of TNF was downregulated in LPS-stimulated RAW264.7 cells with overexpression of Egr2 (Figure 10D). Thus, we further confirmed that although Egr2 was upregulated in M2 macrophages, expression of this protein is also important for the polarization of M1-like macrophages and regulation of expression of M1 markers.

### Overexpression of Egr2 in IFNγ-treated macrophages resulted in upregulation of Egr2, IL-6, and TNF on a protein level

**Figure 11:**
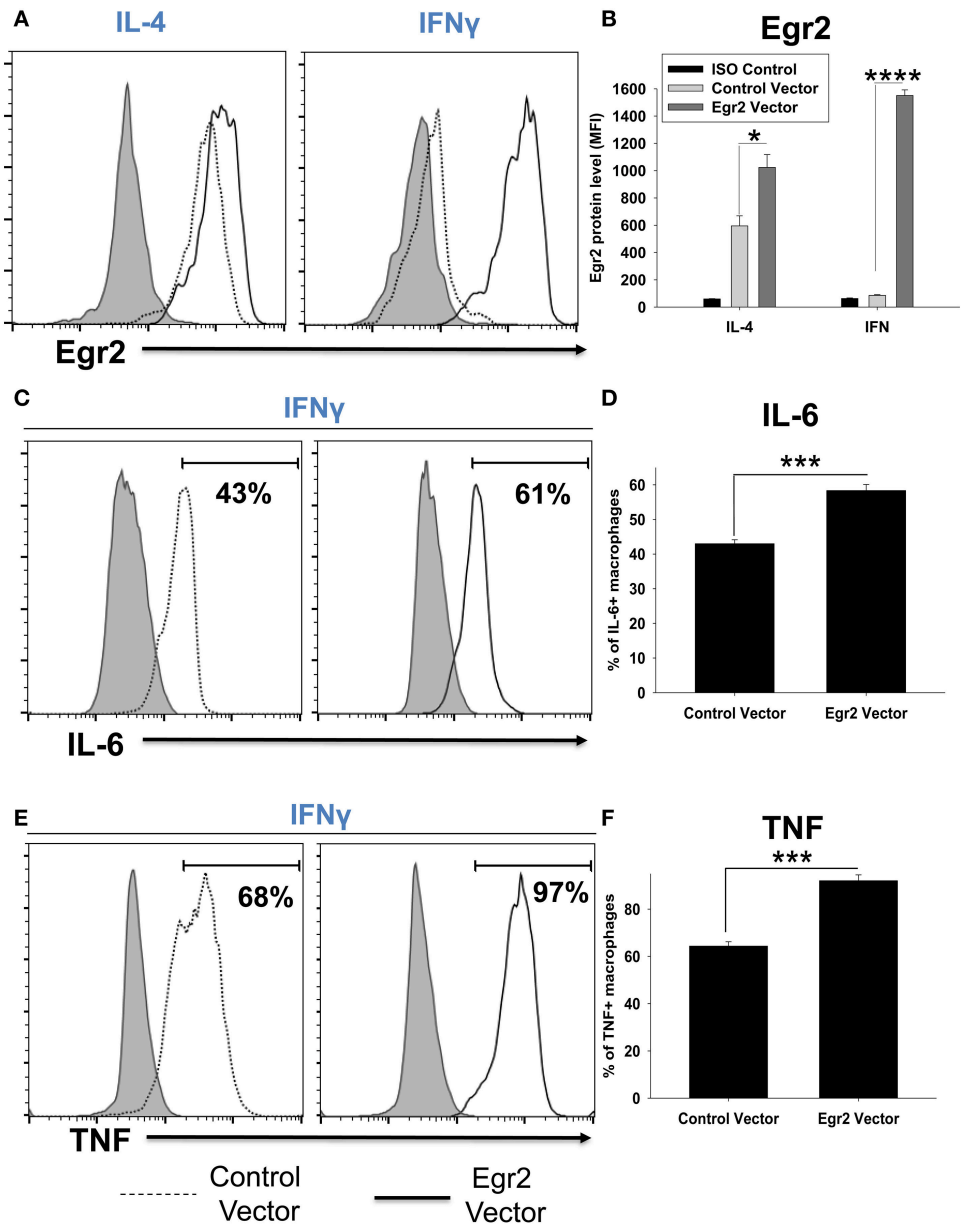
Analysis of expression of Egr2, IL-6, and TNF on a protein level in activated macrophage cell line with overexpression of Egr2. Macrophage cell line RAW264.7 was transfected with empty (control) pMIG plasmid vector (Control Vector), or vector with Egr2 (Egr2 Vector) for 24 h and after which the cells were activated with IL-4 or IFNγ for another 24 h-time period as in Figure 10. The cells were washed, fixed/permeabilized and stained for intracellular Egr2, IL-6, or TNF with fluorescently labeled mAbs as described in *Materials and Methods*. GFP^+^ gated transfected cells were analyzed for expression of Egr2, IL-6, or TNF by three-color flow cytometry. Expression of Egr2 **(A,B)**, IL-6 **(C,D)** and TNF **(E,F)** in cells transfected with Egr2 Vector (solid line) vs. Control Vector (dotted line) are shown on representative histogram graphs **(A,C,D)**. Staining with isotype-matched control mAbs is shown by shaded histograms. Quantitative analysis (mean fluorescence intensity level for Egr2 and percentage of positive cells for IL-6 and TNF) is shown in **(B,D,F)**. In **(B,D,E)**, mean ± S.E. of six separate culture plate wells is shown. Statistically significant differences in the expression levels are shown on each figure (^*^p < 0.05; ^***^p < 0.001; ^****^p < 0.0001).

We validated that transfection of RAW264.7 cells with Egr2 plasmid resulted in upregulation of Egr2 on a protein level using multi-color flow cytometry. We found that expression of Egr2 was significantly increased in F4/80^+^GFP^+^ transfected cells treated with IL-4 or IFNγ (Figures 11A,B). Moreover, the expressions of M1 markers IL-6 (Figures 11C,D) and TNF (Figures 11E,F) were also increased on a protein level in F4/80^+^GFP^+^ transfected cells treated with IFNγ. Thus, we confirmed that expression of Egr2 resulted in upregulation of M1 markers on a protein level.

### Knockdown of Egr2 did not downregulate expressions of SOCS1, SOCS2, and SOCS3 in IFNγ-stimulated macrophages

It has been previously reported that Egr2 directly regulates the expression of SOCS1 and/or SOCS3, but not SOCS2 proteins, in lymphoid and dendritic cells, which are important regulators of pro-inflammatory (IL-6, TNF) and anti-inflammatory (e.g., IL-10) cytokines (20, 21). Here, we verified whether Egr2 regulates the expression of SOCS1, SOCS2, and SOCS3 in activated macrophages. First, we investigated the kinetics of expression of SOCS1, SOCS2, and SOCS3 in M2 and M1 macrophages stimulated with IL-4 or IFNγ, respectively. Similar to the pattern of expression of Egr1, Egr2, and Egr3 proteins, SOCS1 was upregulated by both IL-4, and, to a higher degree, by IFNγ (Figure S5A). SOCS2 was upregulated by IL-4 (Figure S5B), and SOCS3 was upregulated by IFNγ, but not by IL-4 (Figure S5C). However, in contrast to expected results that Egr2 positively regulate SOCSs proteins, knockdown of Egr2 in IFNγ-treated BMDMs resulted in upregulation (not downregulation) of SOCS1, SOCS2, and SOCS3 (Figures S6A–C). Thus, we found that SOCS1, SOCS2, and SOCS3 were not positively regulated by Egr2 in M1-like macrophages.

### Knockdown of Egr2 downregulate expressions of CEBPβ in M0 and M2 macrophages, while overexpression of Egr2 in M1 macrophages upregulate CEBPβ

**Figure 12:**
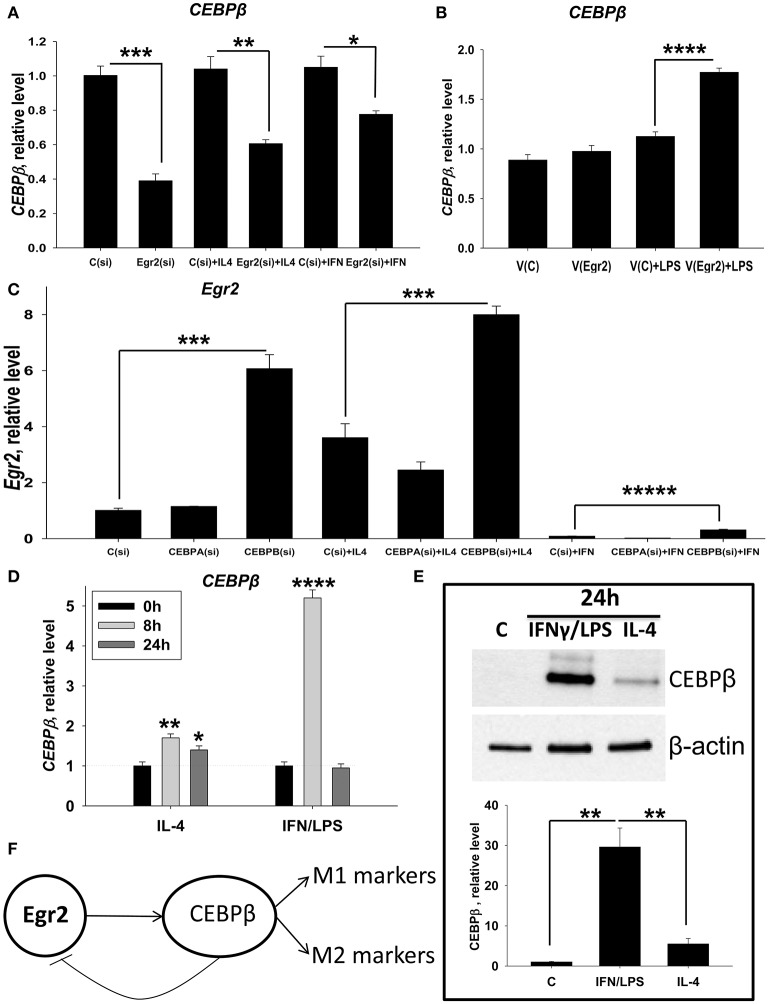
Analysis of reciprocal regulation of CEBPβ and Egr2 in M1 and M2 macrophages. **(A)** Effect of inhibition of Egr2 on CEBPβ expression in M0, M2, and M1 macrophages. Bone-marrow-derived macrophages (BMDMs) were transfected with siRNA cocktail for Egr2 [Egr2(si)] or control siRNA [C(si)] for 24 h as described in *Materials and Methods*, and after which the cells were used as unstimulated [C(si) and Egr2(si)] or activated with IL-4 [C(si) + IL4 and Egr2(si) + IL4] or IFNγ [C(si)+IFN and Egr2(si)+IFN] for another 24 h-time period as in Figure 3. The cells were washed, mRNA was isolated and the expressions of *CEBP*β was analyzed by real-time PCR as described in *Materials and Methods*. **(B)** Effect of overexpression of Egr2 on CEBPβ expression in M1 macrophages. Macrophage cell line RAW264.7 was transfected with pMIG expression vector plasmid or empty plasmid vector [V(C) or vector with Egr2 (V(Egr2)] for 24 h and after which the cells were used as unstimulated [V(C) and V(Egr2)] or activated with LPS for another 24 h-time period [V(C)+LPS and V(Egr2)+LPS] as in Figure 8. The cells were washed, mRNA was isolated and the expressions of *CEBP*β were analyzed by real-time PCR as described in *Materials and Methods*. **(C)** Effect of inhibition of CEBPβ on Egr2 expression in unstimulated (M0), M2, and M1 macrophages. BMDMs were transfected with siRNA cocktail for CEBPα [CEBPA(si)], CEBPβ [CEBPB(si)], or control siRNA [C(si)] for 24 h as described in *Materials and Methods*. After which, the cells were used as unstimulated [C(si), CEBPA(si), and CEBPB(si)] or activated with IL-4 [C(si)+IL4, CEBPA(si)+IL4, and CEBPB(si)+IL4] or IFNγ [C(si)+IFN, CEBPA(si)+IFN and CEBPB(si)+IFN] for another 24 h-time period as in Figure 3. The cells were washed, mRNA was isolated and the expressions of *Egr2* was analyzed by real-time PCR as described in *Materials and Methods*. **(D)** Kinetics of expression of *CEBP*β on mRNA level in M2 (IL-4) and M1 (IFNγ/LPS) macrophages. BMDMs were stimulated with IL-4 or IFNγ/LPS and the levels of *CEBP*β expression was determined by real-time RT PCR immediately (0 h), 8, and 24 h after activation similarly as for Figure 2. Mean ± S.D. of triplicate is shown (^*^p < 0.05; ^**^p < 0.01; ^****^p < 0.0001 when compared to untreated (0 h) cells). **(E)** Expression of CEBPβ on protein level in M2 (IL-4) and M1 (IFNγ/LPS) macrophages. BMDMs were used unstimulated (Cont) or stimulated with IL-4, or IFNγ with LPS for 24 h as for Figure 3 and the level of expression of CEBPβ was analyzed by western blot as described in *Materials and Methods*. A representative blot (upper image) and quantitative analysis (bottom graph) are shown here. The whole blots are shown in Figure S7. **(F)** Model of reciprocal regulation of CEBPβ and Egr2 in M1 and M2 macrophages. In both M1 and M2 macrophages Egr1 upregulate CEBPβ, which in turn induce expression of M1 or M2 markers, respectively. In M1 macrophages, high level of CEBPβ inhibits Egr2 leading to non-responsiveness of these cells to further simulation. In **(A–C,E)**, mean ± S.E. of 4–6 separate culture plate wells is shown (^*^p < 0.05; ^**^p < 0.01; ^***^p < 0.001; ^****^p < 0.0001; ^*****^p < 0.00001).

We further investigated mechanisms by which Egr2 is involved in the upregulated expression of M1 and M2 markers in macrophages. We found that SOCSs molecules are not the target for Egr2 in macrophages (Figures S5, S6). Therefore, we investigated other possible direct targets for Egr2. It has been demonstrated that the Egr family proteins Egr1, Egr2, and Egr3 directly upregulate CEBPβ, one of the major transcription factors in macrophages (37). Many studies have previously shown that CEBPβ is important for macrophage activation and for the induction of expression of both M1 and M2 markers (12–14). Here we investigated whether Egr2 affected the expression of CEBPβ. We found that siRNA for Egr2 downregulated this transcription factor in unstimulated (M0), IL-4 treated (M2), and IFNγ-treated macrophages (Figure 12A). When we upregulated Egr2 in an IFNγ-stimulated M1 macrophage line that exhibited a low level of baseline Egr2 expression, we found that *CEBP*β was upregulated (Figure 12B). Thus, we found that Egr2 positively regulates the expression of CEBPβ in unstimulated and in M1- and M2-stimulated macrophages.

### CEBPβ downregulates the expression of Egr2 in M1-like macrophages

It was previously reported that CEBPβ could negatively regulate the expression of Egr2 (38). We proposed that similar mechanism play a role in M1-like macrophages where Egr2 is significantly downregulated. To test this hypothesis, we investigated whether the expression of Egr2 would be upregulated in unstimulated M0 or stimulated M1 and M2 macrophages where CEBPβ knockdown by siRNA treatment. Expression of Egr2 was upregulated in M0, M2 (IL-4), and M1 (IFNγ) macrophages treated with siRNA for CEBPβ [Figure 12C, *Cebpb(si)*]. When siRNA for CEBPα (as an additional negative control) was employed, Egr2 expression was unaffected [Figure 12C, *Cebpa(si)*]. We also found that siRNA for CEBPβ resulted in a decrease in M1 (NOS2, TNF) and M2 (Arg1, Ym1) markers in IFNγ- or IL-4- stimulated macrophages (not shown), which confirms previous reports. Thus, we found that CEBPβ, but not CEBPα, inhibits the expression of Egr2.

Next, we investigated the level of expression of CEBPβ in unstimulated or M2 (IL-4) or M1 (IFNγ/LPS)-stimulated macrophages. *CEBP*β was upregulated 1.7- and 1.4-fold in M2 macrophages at 8 and 24 h. In M1 macrophages, upregulation of *CEBP*β was quite marked (5-fold) at 8 h, but only transient returning to control level at 24 h (Figure 12D). When we compared the expression of CEBPβ at a protein level, we found that this transcription factor was not detectable in untreated M0 cells. However, CEBPβ was detected in M1- and M2-like macrophages after 24 h of stimulation with IFNγ/LPS or IL-4 (Figure 12E, Figure S7). CEBPβ was expressed at a much higher level in M1-like macrophages compared to M2-like macrophages (Figure 12E, Figure S7), which was correlated with the low level of Egr2 in M1-like macrophages or macrophages with CEBPβ knockdown (Figure 12C). Thus, these data suggest that CEBPβ negatively regulate Egr2 in M1-like macrophages.

## Discussion

In this study, we demonstrated an important role of Egr-family proteins in macrophage activation driven by M1- or M2-like stimuli. We found that Egr2 is expressed in non-activated M0 macrophages, upregulated in M2 macrophages, and significantly downregulated in M1 macrophages where Egr2 expression remained at a low level for an extended period. We also discovered that Egr2 expression was important for the upregulation of both M1 and M2 markers. Our data also indicate that Egr2 expression is important for M0 or M2 macrophages to make the transition to M1, while M1 macrophages exhibited a low level of Egr2 24 h post-stimulation, and respond poorly to M1**-** or M2-like stimuli, and exhibit an Egr2^low^ deactivated phenotype, which is characterized by upregulation of IL-10 (Figure S8).

Mechanistically we found that Egr2 was important for upregulation of CEBPβ in M2 and to less extent of degree M1 macrophages, which in turn, inhibited Egr2 expression in M1 macrophages. These data are in good agreement with the fact that Egr2 is significantly downregulated in M1 but not in M2 cells (Figures 2C,D). Moreover, the peak of expression of Egr2 was at 8 h in M2 macrophages, and after that, the expression of Egr2 was slightly declined at 24 h (Figure 1D), which was consistent with the highest level of *CEBP*β at 8 h and a slight decline at 24 h (Figure 12D). Factor Egr1 could also contribute to the upregulation of CEBPβ in M2 macrophages at 24 h since Egr1 reached the highest level at that time (Figure 1A). In case of M1 cells, CEBPβ could be transiently induced by Egr1 and Egr3 both of which are expressed at the highest level in IFNγ-stimulated macrophages at 8 h and decline by 24 h post-stimulation (Figures 2C,E). Egr2 is also still present in M1 macrophages at 8 h contributing to a high level of mRNA for CEBPβ at that time (Figure 2D). Base on all these data we proposed the model that Egr2 positively regulate CEBPβ that, in turn, promote expression of M1 and M2 markers, while CEBPβ provides a negative regulatory feedback loop downregulating Egr2 (Figure 12F). Thus, taken together our study demonstrate that high level of CEBPβ negatively regulated Egr2 in M1 macrophages.

Although distinct pathways of macrophages activation are recognized, little is known about the regulation of such activation on a molecular and transcriptional level (2). In this study, we demonstrated that Egr2 is involved in the process of activation, polarization, and plasticity of macrophages. Several studies suggest that M1 macrophages exhibit a low level of plasticity *in vivo* (28). Moreover, it was clearly demonstrated that high doses of LPS induce tolerance in macrophages when they decrease the production of pro-inflammatory cytokines IL-1β, IL-6, and TNF and upregulate IL-10 (39). In our study, we found that Egr2^low^ macrophages also downregulated these markers and up-regulated IL-10. Thus, Egr2 may play an important role in LPS tolerance and other types of tolerance in M1-polarized macrophages *in vivo*.

An important question is how Egr2 and CEBPβ mediate macrophages activation in mixed M1/M2 activation when IL-4 and IFNγ/LPS are present at the same time. Since M1 stimuli downregulate Egr2 relatively late (24 h post-activation) and M2 stimuli upregulate Egr2 as early as 3–8 h after stimulation (Figure 1D), we expect that Egr2 and CEBPβ would be first upregulated at 3–8 h and induce expression of M1 and M2 markers at these time-points. At 24 h CEBPβ would downregulate Egr2 in a way similar to that of M1 macrophages (Figure 12). Since both M1 and M2 stimuli upregulate CEBPβ (Figures 12D,E), we expect to see a high level of expression of CEBPβ at 24 h post-activation in M1/M2 mixed activation condition. High level of CEBPβ would downregulate Egr2 in M1/M2-activated macrophages. Thus, we assume that under mixed M1/M2 activation conditions, activated macrophages would express both M1 and M2 markers and would be resistant to further stimulation due to downregulation of Egr2.

Egr2 is involved in the expression of multiple genes in various cells types. In lymphoid cells, it was shown to upregulate expression of SOCS1 and SOCS3 (20). In dendritic cells, conditional knockout of Egr2 abolished expression of SOCS1 (22). In our studies, we did not find downregulation of SOCS1, SOCS2, and SOCS3 in macrophages with knockdown of Egr2. Thus, we believe that it is unlikely that Egr2 mediated this effect through SOCS molecules in activated macrophages. It was demonstrated that Egr2 directly regulated expression of the transcription factors CEBPβ (37). Here, CEBPβ was shown to be important for M1 and M2 polarization. In case of M2 polarization, it is suggested that CEBPβ act through downstream transcription factor PPARγ (2). Our study demonstrates that PPARγ and CEBPβ are regulated by Egr2. Therefore, CEBPβ-PPARγ axis is most likely the mechanism of action of Egr2 in macrophages M2 polarization. For M1 polarization, it was demonstrated that CEBPβ is upregulated in macrophages in response to IFNγ, IL-1, IL-6, TNF, and LPS and induce expression of M1 markers (40). CEBPβ is also required for induction of Th1/Th17-mediated autoimmune neuroinflammation associated with M1/M2 mixed macrophages activation (9, 41). Our study suggests that Egr2 upregulate expression of M1 and M2 markers through activation of CEBPβ and its downstream targets. On the other hand, CEBPβ inhibited Egr2. Since we found that CEBPβ protein is expressed in M1 macrophages at a very high level than in M2 macrophages, this transcription factor appears to inhibit Egr2 in the M1 macrophages causing long-term downregulation of Egr2 and loss of plasticity.

We believe that Egr2 is important for the induction of expression of both M1 and M2 markers (Figures 4,5) since it is expressed in M0 cells, upregulated early in M2 macrophages, and downregulated late in M1 macrophages. Particularly, our study demonstrated that Egr2 was upregulated 3–8 h post-activation in M2 conditions and downregulated only at 24 h post-activation in M1 conditions (Figure 1D). Therefore, we believe that Egr2 is important for both M1 and M2 conditions since Egr2 is expressed at a relatively high level at 3–8 h post-activation in both M1 and M2 macrophages (Figure 1D). We found that most of the M1 markers were upregulated early. For example, M1 marker *NOS2* was upregulated by IFNγ as early as 3 h, peaked at 8 h, and then was slightly decreased by 24 h (Figure 1B). This is consistent with the expression of Egr2 at 3–8 h and downregulation at 24 h in M1 macrophages (Figure 1D).

We believe that Egr2 directly regulate the expression of CEBPβ by binding to its promoter and activating transcription. Our *in silico* analysis revealed that mouse Cebpb promoter area contains ten Egr-binding sites (Figure S9A). In support of this analysis, it was reported that Egr proteins directly induced CEBPβ expression (37). It was also shown that CEBPβ is important for expression of both M1 and M2 markers (12, 14, 34), while we found that CEBPβ is expressed at a very high level in M1 macrophages (Figures 12D,E). Thus, the knockdown of Egr2 in M1 macrophages result in low level of CEBPβ (Figure 12A) leading to low level of M1 markers (Figure 5).

Our study also demonstrated that CEBPβ inhibits Egr2 (Figure 12C). The mechanism for this phenomenon is not as straightforward as positive regulation of CEBPβ by Egr2. We believe that CEBPβ could act alone or together with other co-factors such as Nrf1 to inhibit Egr2 expression by binding to promoter area and repressing Egr2 transcription. It was demonstrated that CEBPβ and Nrf1 efficiently inhibited expression of DSPP gene during odontoblast differentiation by binding to the promoter sequence of this gene (42). In the case of the DSPP gene, both CEBPβ and Nrf1 had the ability to bind to the promoter and repress the transcription acting individually or synergistically by forming complexes with each other. We analyzed Egr2 promoter area and found three potential binding sites for CEBPβ and ten binding sites for Nrf1 (Figure S9B) suggesting the possible involvement of CEBPβ and Nrf1 in Egr2 downregulation. In support of our hypothesis, it was shown that Nrf1 is expressed in macrophages during inflammation *in vivo* and it is upregulated in LPS-treated M1 macrophages *in vitro* at 24–36 h post-treatment (43–45). Moreover, it was shown that Nrf1 in complex with other co-factors (e.g., c-Jun, ATF2) positively regulated TNF expression, a known M1-associated cytokine (46). Thus, the negative transcriptional regulation of Egr2 is likely mediated by CEBPβ alone and/or with the help of other co-factors such as Nrf1.

Our study indicated an important role of Egr2 in macrophages activation *in vivo* during inflammation. The decrease in Egr2 expression inhibited upregulation of MHC class II and CD86, which is very important for antigen presentation and stimulation of CD4 T cells. Our *in vitro* studies also clearly demonstrated that Egr2 is critical for both M1 and M2 types of activation. Thus, in contrast to studies of the role of Egr2 in T/B cells and dendritic cells where Egr2 was found to be a negative regulator of activation via targeting SOCS molecules, Egr2 serve as a positive regulator of macrophage activation via targeting CEBPβ transcription factor but not SOCS molecules. This suggests the potential possibility to target Egr2 in macrophages to inhibit their activation during inflammatory diseases such as autoimmunity. Our study also suggests an importance of having a high level of Egr2 during macrophages reprogramming.

Egr2 belongs to early-immediate response genes, which are induced by multiple stimuli including growth factors, cytokines, hypoxia, and cell stress. The most studied gene from Egr family is Egr1, while the function of Egr2 is less studied (47, 48). There are several conditions such as fibrotic process and hypoxia that affect *in vivo* (49, 50). Therefore it is important to know the level of Egr2 expression in macrophages in such conditions. Indeed, it was demonstrated that Egr2 is upregulated by TGF-β1 during fibrosis, suggesting likely involvement of Egr2 in M2 polarization during the fibrotic process and/or scar formation (51). Hypoxia was shown to upregulate Egr1 and Egr3 and downregulate Egr2 by 45% in unstimulated human monocytes (52). At the same time, hypoxia upregulated both M1 (TNF, IL-6, CD86) and M2 (Arg1) markers in unstimulated human monocytes (52). These data indicate that for unstimulated macrophages hypoxia condition could be viewed as M1/M2 mixed activation stimulus leading to upregulation of M1 and M2 markers and downregulation of Egr2. Another study demonstrated that hypoxia decreased expression of M1 (CD80, CD86, MHC class II, TNF) and M2 (CD206, TREM) markers in LPS- and IL-4- treated human macrophages, respectively (53). This is in line with our data showing that knockdown of Egr2 decreased expression of both M1 and M2 markers (Figures 4, 5).

In our study, we used *in vitro* knockdown/overexpression strategy combined with the adoptive transfer of macrophages during the development of inflammation *in vivo*. Usage of Egr2 knockout mice appeared to be problematic for direct investigation of the role of Egr2 in macrophage activation *in vivo* since Egr2 knockout mice die within a 2-week period after birth (24). It is possible to use irradiation chimeras by doing transplantation of newborn liver hemopoietic stem cells from donor Egr2-deficient mice into lethally irradiated wild-type recipient mice to overcome this problem as was reported earlier (19). However, even in this case *in vivo* experiments investigating macrophage activation during inflammation would be problematic to interpret since Egr2 is also involved in activation and/or development of T/B cells and dendritic cells (20). There is a theoretical possibility to make a conditional knockout of Egr2 only in macrophages using macrophage-specific myeloid-Cre strains crossed with Egr2-loxp animals. However, all current “macrophage-specific” myeloid-Cre systems (CD11b-Cre, F4/80-Cre, LysM-Cre etc.) target also dendritic cells, granulocytes and only a subpopulation of monocytes/macrophages (54). Therefore these systems would be also very problematic to use for *in vivo* experiment with thioglycollate-elicited peritonitis without doing *in vitro* experiments along with adoptive transfer, which we did in the current study. Thus, the direct modulation of Egr2 expression in macrophages *in vivo* is still very challenging, which pose limitations for macrophage reprogramming during pathological conditions in mouse models.

Despite methodological problems, reprogramming of macrophages from M2 to M1 and from M1 to M2 is an important therapeutic ambition in many pathologies. For anti-cancer therapy, it is considered important to reprogram M2 tumor-infiltrating macrophages to M1 (55). Conversely, for many types of inflammatory and autoimmune diseases such as autoimmune neuroinflammation, it is important to reprogram M1 macrophages toward M2 (9). Our study indicates that the second task is likely to be more problematic as it will require the overexpression of Egr2 in addition to stimulation with M2 stimuli to enable the transition of M1 cells to M2. This strategy may be important for future gene therapies of treatment of inflammatory diseases associated with high level of M1 activation such as multiples sclerosis, rheumatoid arthritis, atherosclerosis, sepsis, and others.

## Author contributions

TV and EP conceived the study, planned experiments. TV, AY, and EP conducted experiments. TV, TS, DA, and EP analyzed the data and prepared manuscript.

### Conflict of interest statement

The authors declare that the research was conducted in the absence of any commercial or financial relationships that could be construed as a potential conflict of interest.

## Supplementary material

Figure S1 Analysis of expression of Egr2 in IL-4- and LPS-treated macrophages on a protein level. Bone-marrow-derived macrophages (BMDMs) were treated with IL-4 for 24 h **(A,B)** or LPS **(C)** for 72 h as described in *Materials and Methods* and the cells were analyzed as untreated (Control) or after 24 h, 48 h, and 72 h of incubation with LPS (100 ng/ml). For analysis, the cells were washed, and the expression of Egr2 was analyzed on a protein level by western blot as described in *Material and Methods*. A representative images are shown in **(A,C)**. Quantitative analysis of relative expression levels of Egr2 normalized to β-Actin is shown in **(B)**. Mean ± S.E. of three experiments is shown (^**^*p* < 0.01).

Figure S2 Analysis of expression of Egr2 protein in unstimulated and IL-4-treated macrophages with knockdown of Egr2. Bone-marrow-derived macrophages (BMDMs) were transfected with siRNA cocktail for Egr2 [Egr2(si)] or control siRNA [C(si)] for 24 h as described in *Materials and Methods*, and after which the cells were used as unstimulated [C(si) and Egr2(si)] or activated with IL-4 for another 24 h-time period [C(si)+IL4 and Egr2(si)+IL4] as in Figure 3. The expression of Egr2 was analyzed by western blot as described in *Materials and Methods*. Quantitative analysis of relative expression levels of Egr2 normalized to β-Actin is shown. Representative images are shown in Figure S3. Mean ± S.E. of three separate experiments is shown (^**^*p* < 0.01).

Figure S3 Analysis of expression of Egr2 in unstimulated and IL-4-treated macrophages with knockdown of Egr2. Bone-marrow-derived macrophages (BMDMs) were transfected with siRNA cocktail for Egr2 [Egr2(si)] or control siRNA [C(si)] for 24 h as described in *Materials and Methods*, and after which the cells were used as unstimulated [C(si) and Egr2(si)] or activated with IL-4 for another 24 h-time period [C(si)+IL4 and Egr2(si)+IL4] as in Figure 3. The expression of Egr2 was analyzed by western blot as described in *Materials and Methods*. A representative image of whole blots for Egr2 and β-Actin are shown with marked relevant samples.

Figure S4 Analysis of expression of miR-155 in IFNγ-treated macrophages with knockdown of Egr2. Bone-marrow-derived macrophages (BMDMs) were transfected with siRNA cocktail for Egr2 [Egr2(si)] or control siRNA [C(si)] for 24 h as described in *Materials and Methods*, and after which the cells were used as unstimulated [C(si) and Egr2(si)] or activated with IFNγ for another 24 h-time period [C(si)+IFN and Egr2(si)+IFN] as in Figure 3. The cells were washed, mRNA was isolated and the expression of miR-155 was analyzed by real-time PCR as described in *Materials and Methods*. Mean ± S.E. of six separate culture plate wells is shown (^****^*p* < 0.0001).

Figure S5 Kinetics of expression of SOCS1, SOCS2, and SOCS in macrophages polarized toward M2 with IL-4 or M1 with IFNγ. Bone-marrow-derived macrophages (BMDMs) were analyzed untreated (Control) or treated with IL-4 (IL4) or IFNγ (IFN-γ) as described in *Materials and Methods* and the cells were analyzed after 3, 5, 8, and 24 h of incubation. For analysis, the cells were washed, mRNA was isolated and the expressions of *SOCS1* **(A)**, *SOCS2* **(B)**, and *SOCS3* **(C)** were analyzed by real-time RT PCR as described in *Materials and Methods*. Mean ± S.E. of six separate culture plate wells is shown.

Figure S6 Effect of inhibition of Egr2 on the regulation of SOCS1, SOCS2 and SOCS3 expressions in M2- and M-like macrophages. Bone-marrow-derived macrophages (BMDMs) were transfected with siRNA cocktail for Egr2 [Egr2(si)] or control siRNA [C(si)] for 24 h as described in *Materials and Methods*, and after which, the cells were used as unstimulated [C(si) and Egr2(si)] or activated with IL-4 [C(si)+IL4 and Egr2(si)+IL4] or IFNγ [C(si)+IFN and Egr2(si)+IFN] for another 24 h-time period as in Figure 3. The cells were washed, mRNA was isolated and the expressions of *SOCS1* **(A)**, *SOCS2* **(B)**, and *SCOS3* **(C)** were analyzed by real-time PCR as described in *Materials and Methods*. In **(A–C)**, mean ± S.E. of six separate culture plate wells is shown (^*^*p* < 0.05; ^**^, *p* < 0.01; ^***^*p* < 0.001).

Figure S7 Analysis of the expression of the CEBPβ protein in M2/M(IL-4) and M1/M(FNγ/LPS) macrophages. Bone-marrow-derived macrophages (BMDMs) were used as unstimulated (M0) or stimulated with IFNγ and LPS (M1), or IL-4 (M2) for 24 h as for Figure 12E and the level of expression of CEBPβ was analyzed by western blot as described in *Materials and Methods*. Representative whole blots for CEBPβ and β-Actin are shown.

Figure S8 Model of regulation of macrophages plasticity by Egr2. Unstimulated M0 macrophages express Egr2 and do not express M1 and M2 markers such as NOS2 and Agr1 exhibiting Egr2^+^NOS2^−^Arg1^−^ phenotype. After stimulation with IFNγ and/or LPS macrophages downregulate Egr2 and become M1 with Egr2^−^NOS2^+^Arg1^−^ phenotype. Further stimulation of M1 macrophages with IL-4 or IFNγ/LPS result in deactivated (M-dea) Egr2^−^IL-10^+^ phenotype with a low level of M1 and M2 markers. On the other hand, M0 macrophages stimulated with IL-4 toward M2 become Egr2^+++^NOS2^−^Arg1^+^. Further stimulation of M2 macrophages with IFNγ/LPS result in M1 phenotype with upregulation of M1 and downregulation of M2 markers.

Figure S9 *In silico* analysis of Cebpb gene promoter area for the presence of Egr2-binding sites, and the analysis of Egr2 promoter area for the presence of CEBPβ- and Nrf1- binding sites. **(A)** Mapping of 3,000 bp promoter region upstream of mouse Cebpb gene (chromosome 2) using MULAN software (https://mulan.dcode.org/). Egr-binding sites upstream of Cebpb gene are shown by red boxes. **(B)** Mapping of 3,000 bp promoter region upstream of mouse Egr2 gene (chromosome 10) using MULAN software (https://mulan.dcode.org/). CEBPβ- and Nrf1- binding sites upstream of Egr2 are shown by yellow and green boxes, respectively.
