# Corrigendum: Early Growth Response Gene-2 Is Essential for M1 and M2 Macrophage Activation and Plasticity by Modulation of the Transcription Factor CEBPβ

**DOI:** 10.3389/fimmu.2018.02923

**Published:** 2018-12-13

**Authors:** Tatyana Veremeyko, Amanda W. Y. Yung, Daniel C. Anthony, Tatyana Strekalova, Eugene D. Ponomarev

**Affiliations:** ^1^Faculty of Medicine, School of Biomedical Sciences, The Chinese University of Hong Kong, Shatin, Hong Kong; ^2^Department of Pharmacology, University of Oxford, Oxford, United Kingdom; ^3^Department of Neuroscience, Maastricht University, Maastricht, Netherlands; ^4^Institute of General Pathology and Pathophysiology, Moscow, Russia; ^5^Laboratory of Psychiatric Neurobiology, Institute of Molecular Medicine and Department of Normal Physiology, Sechenov First Moscow State Medical University, Moscow, Russia; ^6^Kunming Institute of Zoology-Chinese University of Hong Kong Joint Laboratory of Bioresources and Molecular Research of Common Diseases, Kunming, China

**Keywords:** CEBPβ, Egr2, M1/M2 balance, activation, inflammation, monocytes/macrophages, plasticity

In the published article, there was an error regarding the affiliation for Tatyana Strekalova. As well as having affiliations 3 and 4, she should also have “Laboratory of Psychiatric Neurobiology, Institute of Molecular Medicine and Department of Normal Physiology, Sechenov First Moscow State Medical University, Moscow, Russia”.

The authors apologize for this error and state that this does not change the scientific conclusions of the article in any way. The original article has been updated.

