# Supplementary figures and images for: Early Growth Response Gene-2 Is Essential for M1 and M2 Macrophage Activation and Plasticity by Modulation of the Transcription Factor CEBPβ
Source: Front Immunol. 2018 Nov 1;9:2515. doi: 10.3389/fimmu.2018.02515 (PMC6221966; doi:10.3389/fimmu.2018.02515)

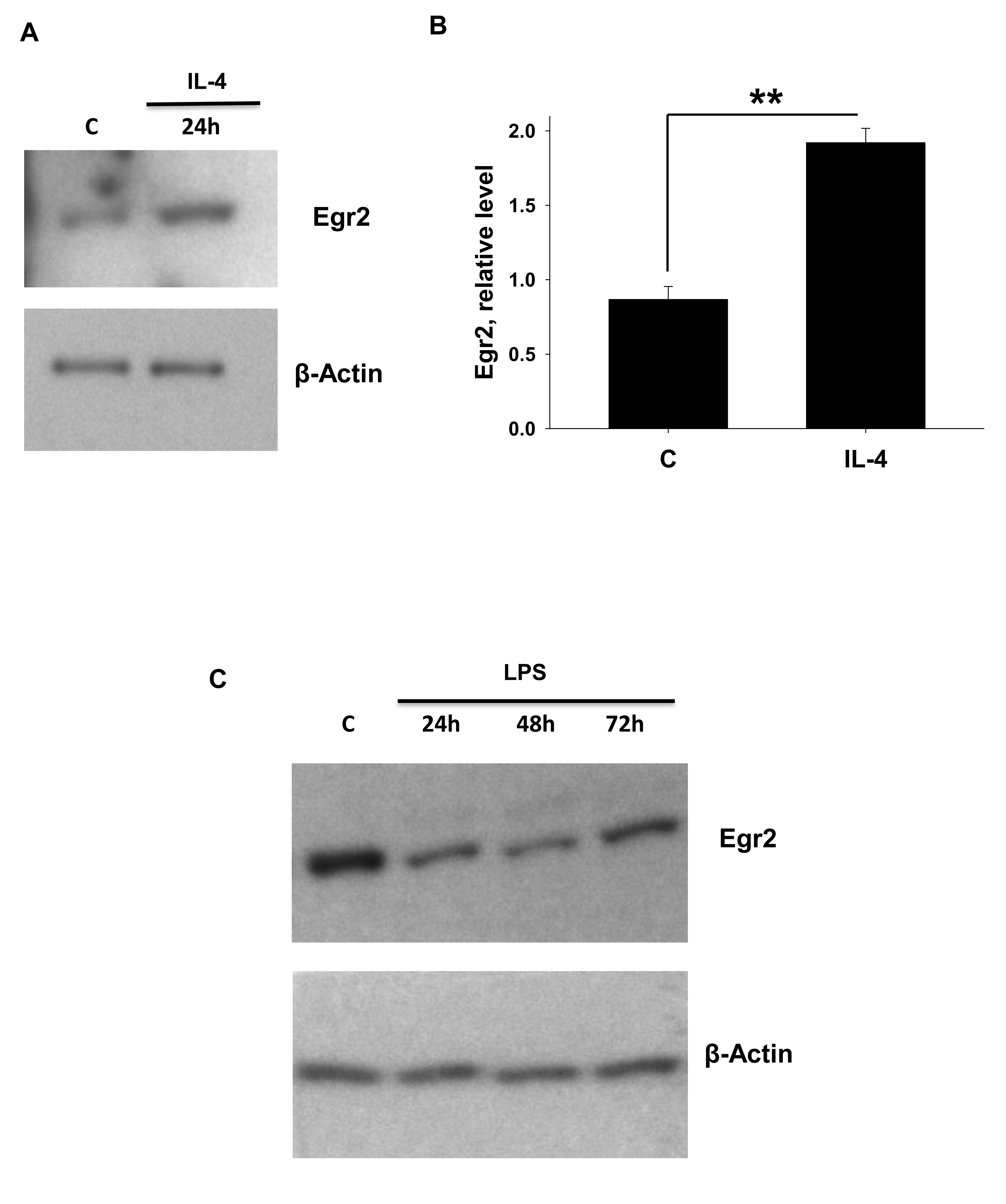

Supplement: Figure S1 — Analysis of expression of Egr2 in IL-4- and LPS-treated macrophages on a protein level. Bone-marrow-derived macrophages (BMDMs) were treated with IL-4 for 24 h (A,B) or LPS (C) for 72 h as described in Materials and Methods and the cells were analyzed as untreated (Control) or after 24 h, 48 h, and 72 h of incubation with LPS (100 ng/ml). For analysis, the cells were washed, and the expression of Egr2 was analyzed on a protein level by western blot as described in Material and Methods. A representative images are shown in (A,C). Quantitative analysis of relative expression levels of Egr2 normalized to β-Actin is shown in (B). Mean ± S.E. of three experiments is shown (**p < 0.01). [file Image_1.jpg]

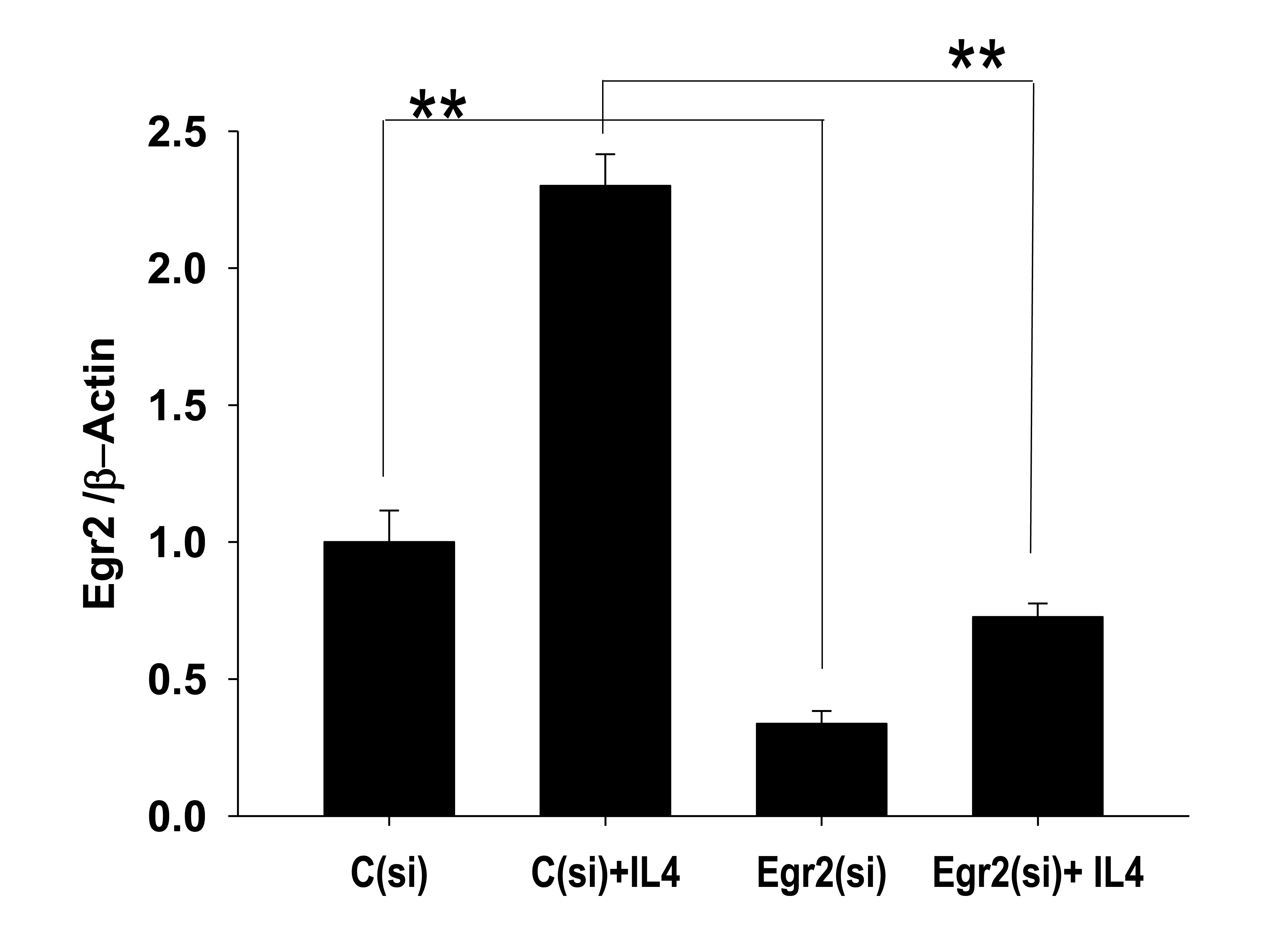

Supplement: Figure S2 — Analysis of expression of Egr2 protein in unstimulated and IL-4-treated macrophages with knockdown of Egr2. Bone-marrow-derived macrophages (BMDMs) were transfected with siRNA cocktail for Egr2 [Egr2(si)] or control siRNA [C(si)] for 24 h as described in Materials and Methods, and after which the cells were used as unstimulated [C(si) and Egr2(si)] or activated with IL-4 for another 24 h-time period [C(si)+IL4 and Egr2(si)+IL4] as in Figure 3. The expression of Egr2 was analyzed by western blot as described in Materials and Methods. Quantitative analysis of relative expression levels of Egr2 normalized to β-Actin is shown. Representative images are shown in Figure S3. Mean ± S.E. of three separate experiments is shown (**p < 0.01). [file Image_2.jpg]

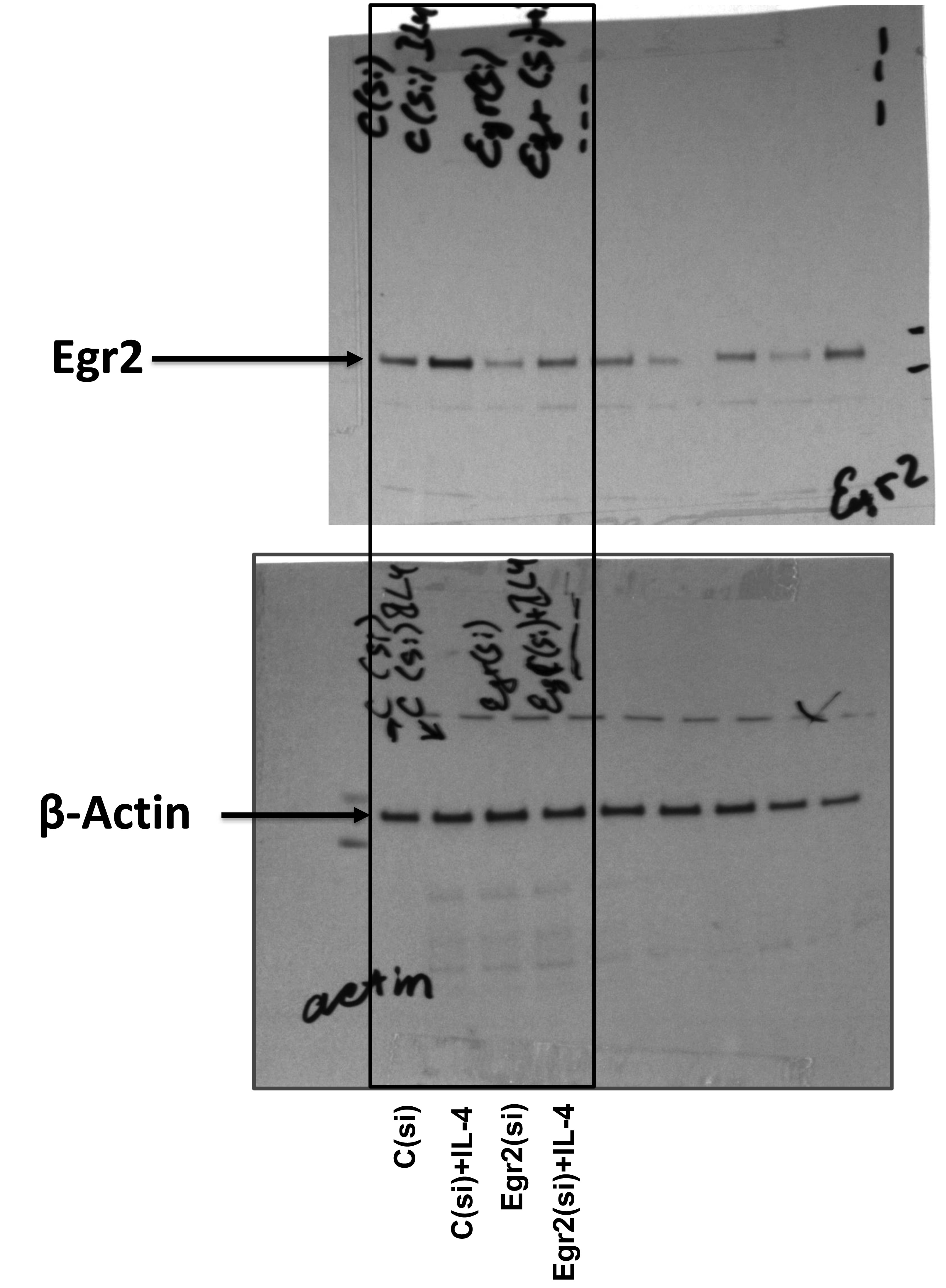

Supplement: Figure S3 — Analysis of expression of Egr2 in unstimulated and IL-4-treated macrophages with knockdown of Egr2. Bone-marrow-derived macrophages (BMDMs) were transfected with siRNA cocktail for Egr2 [Egr2(si)] or control siRNA [C(si)] for 24 h as described in Materials and Methods, and after which the cells were used as unstimulated [C(si) and Egr2(si)] or activated with IL-4 for another 24 h-time period [C(si)+IL4 and Egr2(si)+IL4] as in Figure 3. The expression of Egr2 was analyzed by western blot as described in Materials and Methods. A representative image of whole blots for Egr2 and β-Actin are shown with marked relevant samples. [file Image_3.jpg]

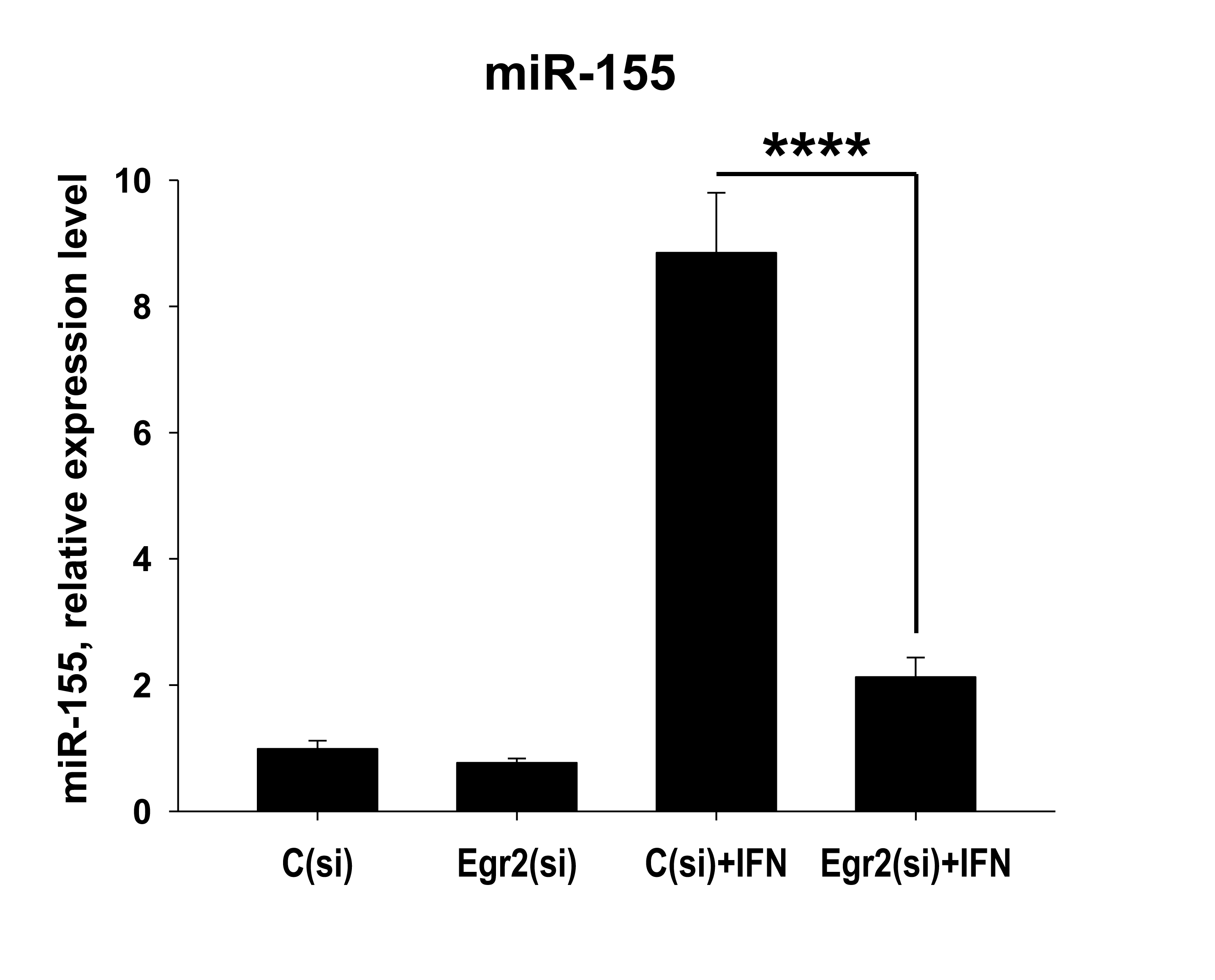

Supplement: Figure S4 — Analysis of expression of miR-155 in IFNγ-treated macrophages with knockdown of Egr2. Bone-marrow-derived macrophages (BMDMs) were transfected with siRNA cocktail for Egr2 [Egr2(si)] or control siRNA [C(si)] for 24 h as described in Materials and Methods, and after which the cells were used as unstimulated [C(si) and Egr2(si)] or activated with IFNγ for another 24 h-time period [C(si)+IFN and Egr2(si)+IFN] as in Figure 3. The cells were washed, mRNA was isolated and the expression of miR-155 was analyzed by real-time PCR as described in Materials and Methods. Mean ± S.E. of six separate culture plate wells is shown (****p < 0.0001). [file Image_4.jpg]

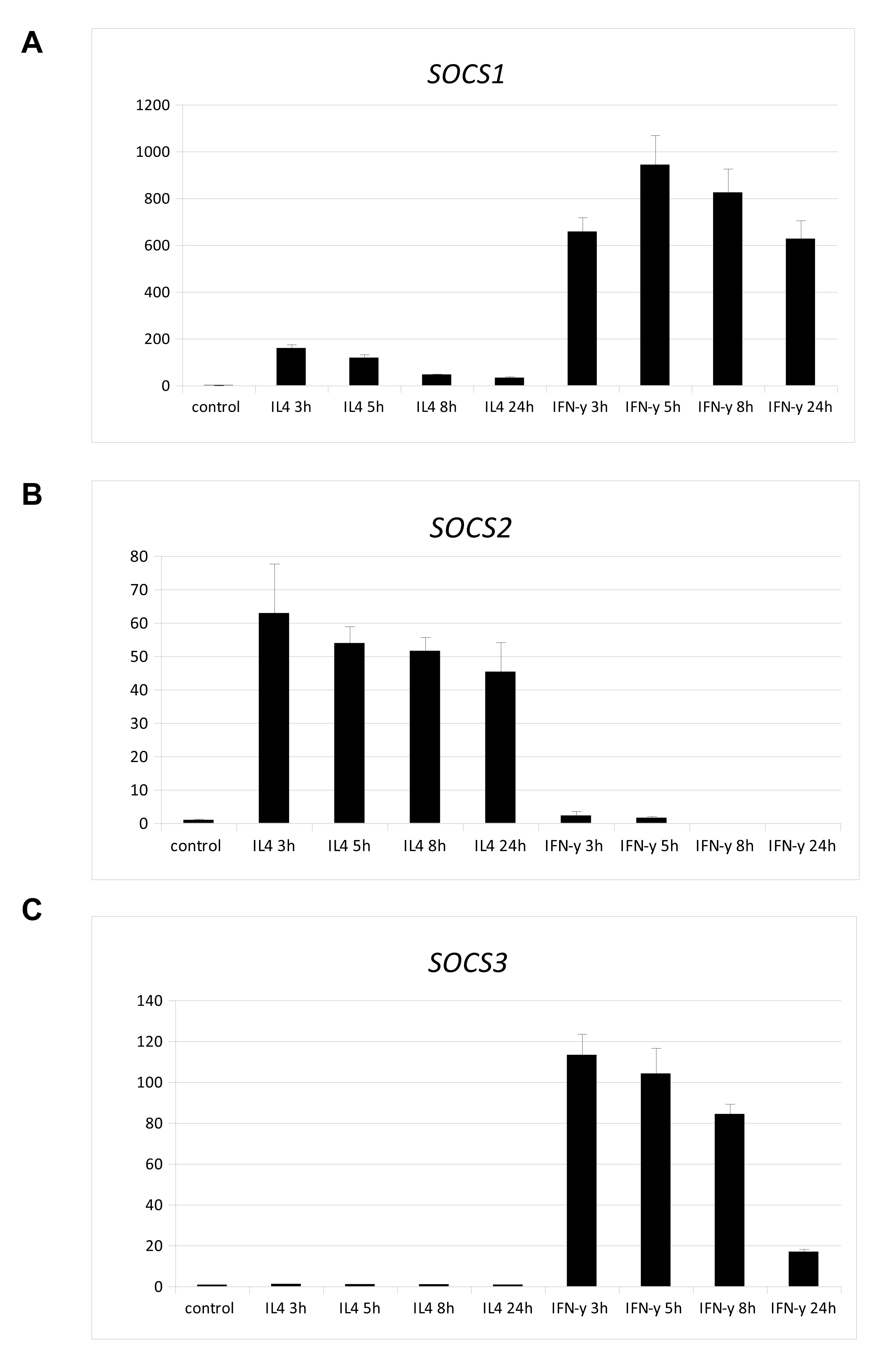

Supplement: Figure S5 — Kinetics of expression of SOCS1, SOCS2, and SOCS in macrophages polarized toward M2 with IL-4 or M1 with IFNγ. Bone-marrow-derived macrophages (BMDMs) were analyzed untreated (Control) or treated with IL-4 (IL4) or IFNγ (IFN-γ) as described in Materials and Methods and the cells were analyzed after 3, 5, 8, and 24 h of incubation. For analysis, the cells were washed, mRNA was isolated and the expressions of SOCS1 (A), SOCS2 (B), and SOCS3 (C) were analyzed by real-time RT PCR as described in Materials and Methods. Mean ± S.E. of six separate culture plate wells is shown. [file Image_5.jpg]

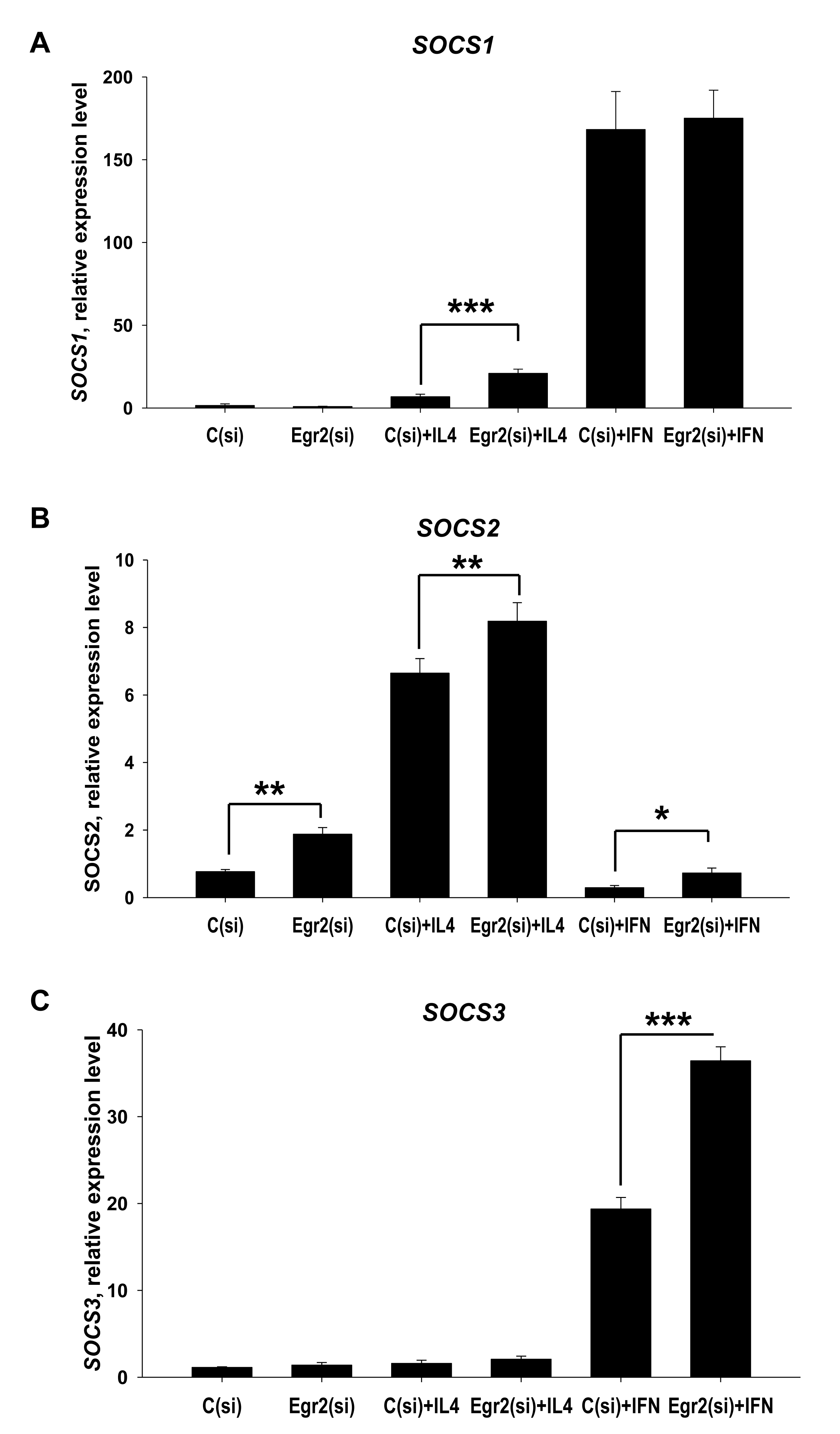

Supplement: Figure S6 — Effect of inhibition of Egr2 on the regulation of SOCS1, SOCS2 and SOCS3 expressions in M2- and M-like macrophages. Bone-marrow-derived macrophages (BMDMs) were transfected with siRNA cocktail for Egr2 [Egr2(si)] or control siRNA [C(si)] for 24 h as described in Materials and Methods, and after which, the cells were used as unstimulated [C(si) and Egr2(si)] or activated with IL-4 [C(si)+IL4 and Egr2(si)+IL4] or IFNγ [C(si)+IFN and Egr2(si)+IFN] for another 24 h-time period as in Figure 3. The cells were washed, mRNA was isolated and the expressions of SOCS1 (A), SOCS2 (B), and SCOS3 (C) were analyzed by real-time PCR as described in Materials and Methods. In (A–C), mean ± S.E. of six separate culture plate wells is shown (*p < 0.05; **, p < 0.01; ***p < 0.001). [file Image_6.jpg]

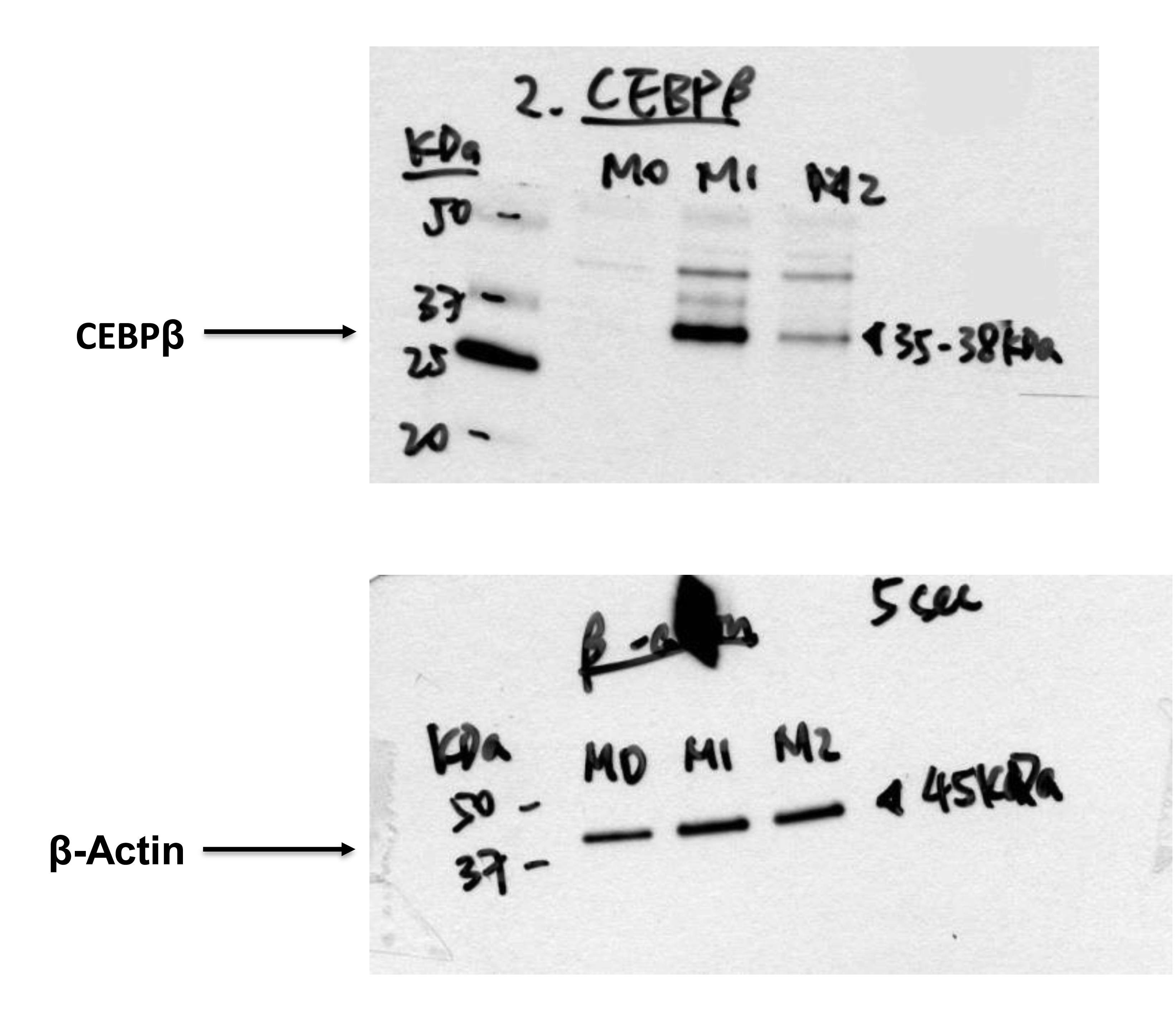

Supplement: Figure S7 — Analysis of the expression of the CEBPβ protein in M2/M(IL-4) and M1/M(FNγ/LPS) macrophages. Bone-marrow-derived macrophages (BMDMs) were used as unstimulated (M0) or stimulated with IFNγ and LPS (M1), or IL-4 (M2) for 24 h as for Figure 12E and the level of expression of CEBPβ was analyzed by western blot as described in Materials and Methods. Representative whole blots for CEBPβ and β-Actin are shown. [file Image_7.jpg]

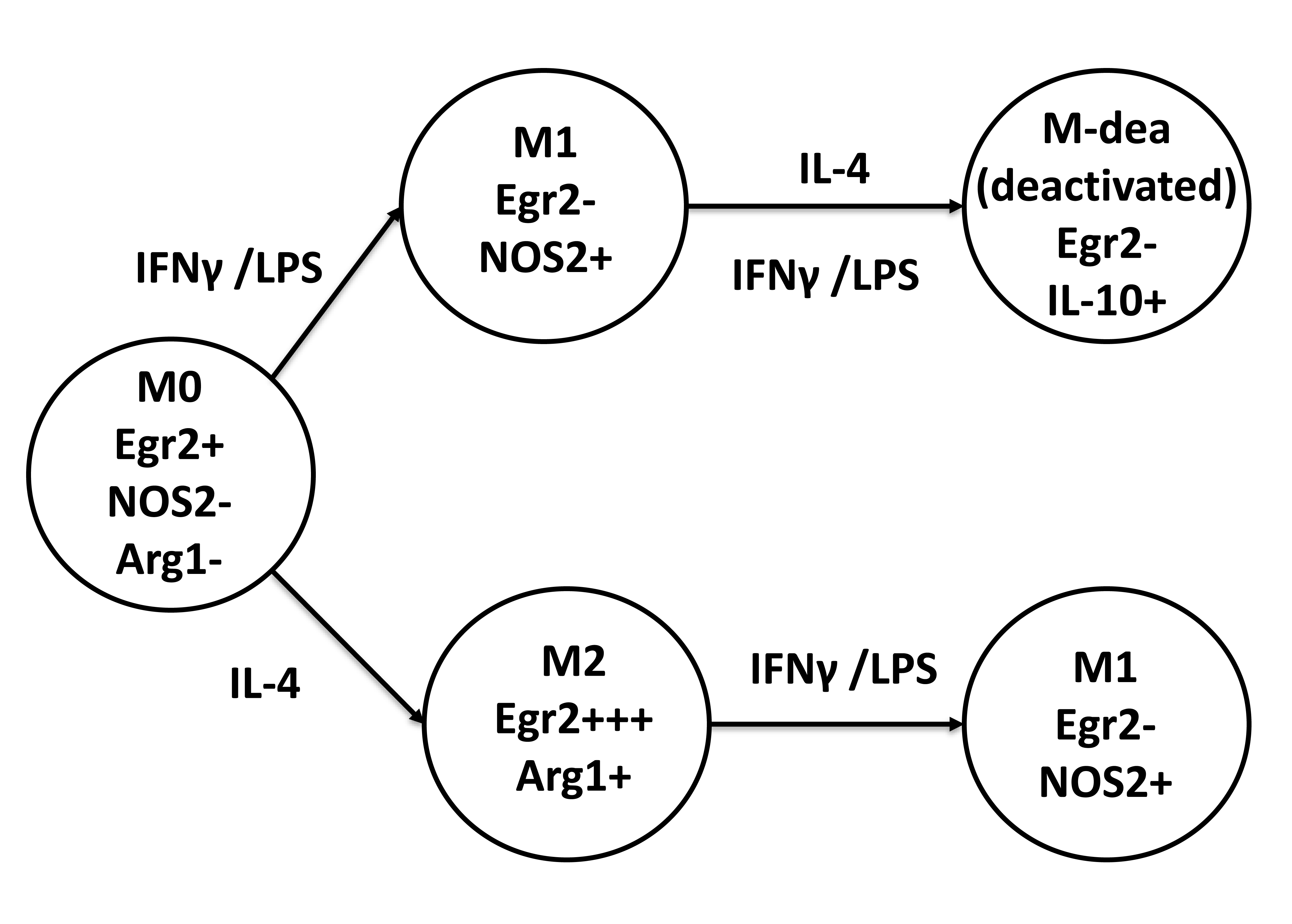

Supplement: Figure S8 — Model of regulation of macrophages plasticity by Egr2. Unstimulated M0 macrophages express Egr2 and do not express M1 and M2 markers such as NOS2 and Agr1 exhibiting Egr2+NOS2−Arg1− phenotype. After stimulation with IFNγ and/or LPS macrophages downregulate Egr2 and become M1 with Egr2−NOS2+Arg1− phenotype. Further stimulation of M1 macrophages with IL-4 or IFNγ/LPS result in deactivated (M-dea) Egr2−IL-10+ phenotype with a low level of M1 and M2 markers. On the other hand, M0 macrophages stimulated with IL-4 toward M2 become Egr2+++NOS2−Arg1+. Further stimulation of M2 macrophages with IFNγ/LPS result in M1 phenotype with upregulation of M1 and downregulation of M2 markers. [file Image_8.jpg]

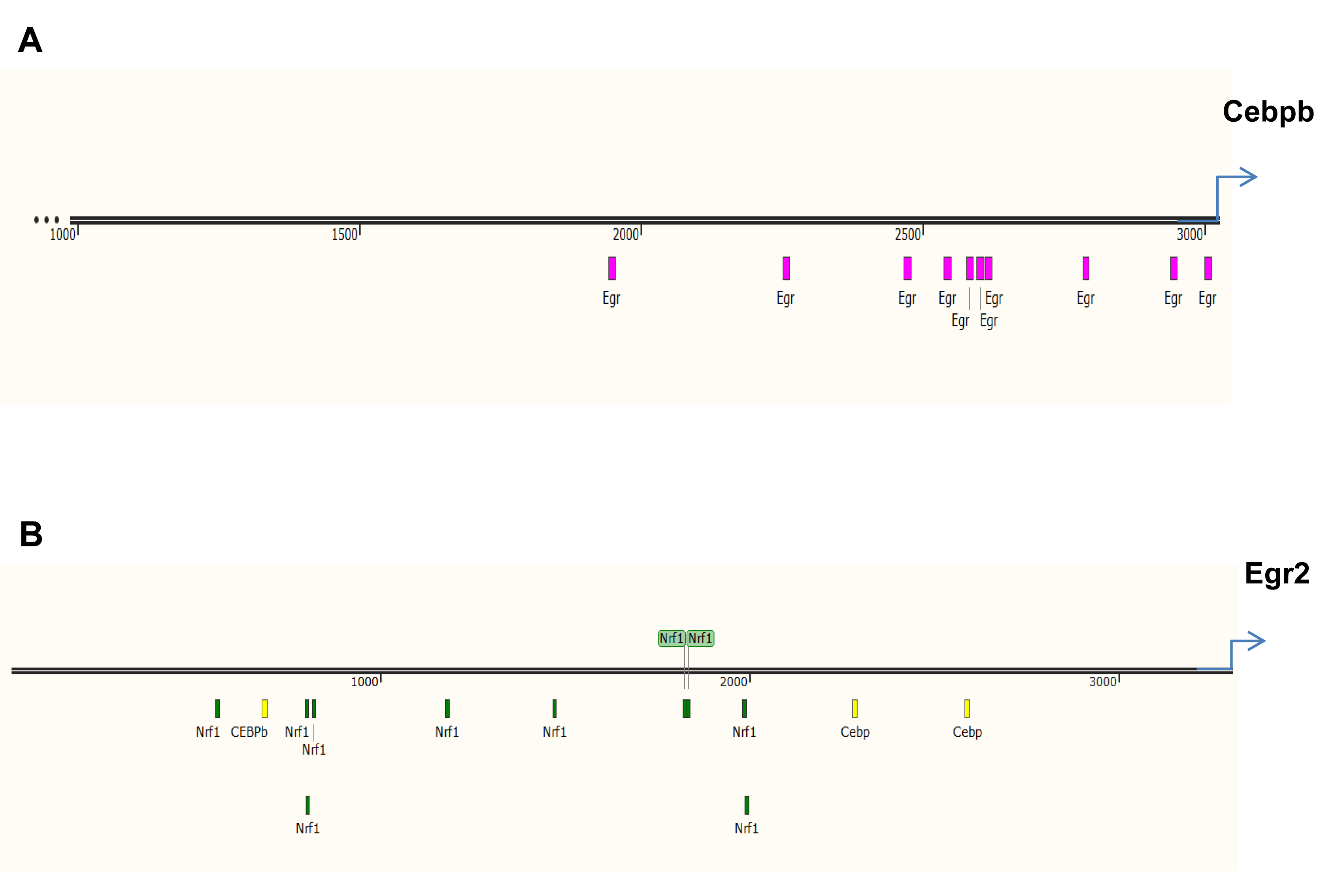

Supplement: Figure S9 — In silico analysis of Cebpb gene promoter area for the presence of Egr2-binding sites, and the analysis of Egr2 promoter area for the presence of CEBPβ- and Nrf1- binding sites. (A) Mapping of 3,000 bp promoter region upstream of mouse Cebpb gene (chromosome 2) using MULAN software (https://mulan.dcode.org/). Egr-binding sites upstream of Cebpb gene are shown by red boxes. (B) Mapping of 3,000 bp promoter region upstream of mouse Egr2 gene (chromosome 10) using MULAN software (https://mulan.dcode.org/). CEBPβ- and Nrf1- binding sites upstream of Egr2 are shown by yellow and green boxes, respectively. [file Image_9.jpg]
